# Temporal changes in SARS-CoV-2 clearance kinetics and the optimal design of antiviral pharmacodynamic studies: an individual patient data meta-analysis of a randomised, controlled, adaptive platform study (PLATCOV)

**DOI:** 10.1016/S1473-3099(24)00183-X

**Published:** 2024-04-24

**Authors:** Phrutsamon Wongnak, William H K Schilling, Podjanee Jittamala, Simon Boyd, Viravarn Luvira, Tanaya Siripoon, Thundon Ngamprasertchai, Elizabeth M Batty, Shivani Singh, Jindarat Kouhathong, Watcharee Pagornrat, Patpannee Khanthagan, Borimas Hanboonkunupakarn, Kittiyod Poovorawan, Mayfong Mayxay, Kesinee Chotivanich, Mallika Imwong, Sasithon Pukrittayakamee, Elizabeth A Ashley, Arjen M Dondorp, Nicholas P J Day, Mauro M Teixeira, Watcharapong Piyaphanee, Weerapong Phumratanaprapin, Nicholas J White, James A Watson, on behalf of the PLATCOV Collaborative Group

**Affiliations:** Mahidol Oxford Tropical Medicine Research Unit, Mahidol University, Bangkok, Thailand; Mahidol Oxford Tropical Medicine Research Unit, Mahidol University, Bangkok, Thailand; Centre for Tropical Medicine and Global Health, University of Oxford, Oxford, UK; Mahidol Oxford Tropical Medicine Research Unit, Mahidol University, Bangkok, Thailand; Department of Tropical Hygiene, Mahidol University, Bangkok, Thailand; Mahidol Oxford Tropical Medicine Research Unit, Mahidol University, Bangkok, Thailand; Centre for Tropical Medicine and Global Health, University of Oxford, Oxford, UK; Department of Clinical Tropical Medicine, Mahidol University, Bangkok, Thailand; Department of Clinical Tropical Medicine, Mahidol University, Bangkok, Thailand; Department of Tropical Hygiene Mahidol University, Bangkok, Thailand; Mahidol Oxford Tropical Medicine Research Unit, Mahidol University, Bangkok, Thailand; Centre for Tropical Medicine and Global Health, University of Oxford, Oxford, UK; Mahidol Oxford Tropical Medicine Research Unit, Mahidol University, Bangkok, Thailand; Centre for Tropical Medicine and Global Health, University of Oxford, Oxford, UK; Mahidol Oxford Tropical Medicine Research Unit, Mahidol University, Bangkok, Thailand; Mahidol Oxford Tropical Medicine Research Unit, Mahidol University, Bangkok, Thailand; Mahidol Oxford Tropical Medicine Research Unit, Mahidol University, Bangkok, Thailand; Mahidol Oxford Tropical Medicine Research Unit, Mahidol University, Bangkok, Thailand; Department of Clinical Tropical Medicine, Mahidol University, Bangkok, Thailand; Mahidol Oxford Tropical Medicine Research Unit, Mahidol University, Bangkok, Thailand; Department of Clinical Tropical Medicine, Mahidol University, Bangkok, Thailand; Centre for Tropical Medicine and Global Health, University of Oxford, Oxford, UK; Lao-Oxford-Mahosot Hospital-Wellcome Trust Research Unit, Mahosot Hospital, Vientiane, Laos; Institute for Research and Education Development, University of Health Sciences, Vientiane, Laos; Mahidol Oxford Tropical Medicine Research Unit, Mahidol University, Bangkok, Thailand; Department of Clinical Tropical Medicine, Mahidol University, Bangkok, Thailand; Mahidol Oxford Tropical Medicine Research Unit, Mahidol University, Bangkok, Thailand; Department of Molecular Tropical Medicine and Genetics, Mahidol University, Bangkok, Thailand; Mahidol Oxford Tropical Medicine Research Unit, Mahidol University, Bangkok, Thailand; Department of Clinical Tropical Medicine, Mahidol University, Bangkok, Thailand; Centre for Tropical Medicine and Global Health, University of Oxford, Oxford, UK; Lao-Oxford-Mahosot Hospital-Wellcome Trust Research Unit, Mahosot Hospital, Vientiane, Laos; Mahidol Oxford Tropical Medicine Research Unit, Mahidol University, Bangkok, Thailand; Centre for Tropical Medicine and Global Health, University of Oxford, Oxford, UK; Mahidol Oxford Tropical Medicine Research Unit, Mahidol University, Bangkok, Thailand; Clinical Research Unit, Center for Advanced and Innovative Therapies, Universidade Federal de Minas Gerais, Brazil; Department of Clinical Tropical Medicine Mahidol University, Bangkok, Thailand; Department of Clinical Tropical Medicine Mahidol University, Bangkok, Thailand; Mahidol Oxford Tropical Medicine Research Unit, Mahidol University, Bangkok, Thailand; Centre for Tropical Medicine and Global Health, University of Oxford, Oxford, UK; Centre for Tropical Medicine and Global Health, University of Oxford, Oxford, UK; Oxford University Clinical Research Unit, Hospital for Tropical Diseases, Ho Chi Minh City, Vietnam

## Abstract

**Background:**

Effective antiviral drugs prevent hospitalisation and death from COVID-19. Antiviral efficacy can be efficiently assessed in vivo by measuring rates of SARS-CoV-2 clearance estimated from serial viral genome densities quantitated in nasopharyngeal or oropharyngeal swab eluates. We conducted an individual patient data meta-analysis of unblinded arms in the PLATCOV platform trial to characterise changes in viral clearance kinetics and infer optimal design and interpretation of antiviral pharmacometric evaluations.

**Methods:**

Serial viral density data were analysed from symptomatic, previously healthy, adult patients (within 4 days of symptom onset) enrolled in a large multicentre, randomised, adaptive, pharmacodynamic, platform trial (PLATCOV) comparing antiviral interventions for SARS-CoV-2. Viral clearance rates over 1 week were estimated under a hierarchical Bayesian linear model with B-splines used to characterise temporal changes in enrolment viral densities and clearance rates. Bootstrap re-sampling was used to assess the optimal duration of follow-up for pharmacometric assessment, where optimal was defined as maximising the expected Z score when comparing effective antivirals with no treatment. PLATCOV is registered at ClinicalTrials.gov, NCT05041907.

**Findings:**

Between Sept 29, 2021, and Oct 20, 2023, 1262 patients were randomly assigned in the PLATCOV trial. Unblinded data were available from 800 patients (who provided 16 818 oropharyngeal viral quantitative PCR [qPCR] measurements), of whom 504 (63%) were female. 783 (98%) patients had received at least one vaccine dose and 703 (88%) were fully vaccinated. SARS-CoV-2 viral clearance was biphasic (bi-exponential). The first phase (α) was accelerated by effective interventions. For all the effective interventions studied, maximum discriminative power (maximum expected Z score) was obtained when evaluating serial data from the first 5 days after enrolment. Over the 2-year period studied, median viral clearance half-lives estimated over 7 days shortened from 16·6 h (IQR 15·3 to 18·2) in September, 2021, to 9·2 h (8·0 to 10·6) in October, 2023, in patients receiving no antiviral drugs, equivalent to a relative reduction of 44% (95% credible interval [CrI] 19 to 64). A parallel reduction in viral clearance half-lives over time was observed in patients receiving antiviral drugs. For example, in the 158 patients assigned to ritonavir-boosted nirmatrelvir (3380 qPCR measurements), the median viral clearance half-life reduced from 6·4 h (IQR 5·7 to 7·3) in June, 2022, to 4·8 h (4·2 to 5·5) in October, 2023, a relative reduction of 26% (95% CrI –4 to 42).

**Interpretation:**

SARS-CoV-2 viral clearance kinetics in symptomatic, vaccinated individuals accelerated substantially over 2 years of the pandemic, necessitating a change to how new SARS-CoV-2 antivirals are compared (ie, shortening the period of pharmacodynamic assessment). As of writing (October, 2023), antiviral efficacy in COVID-19 can be efficiently assessed in vivo using serial qPCRs from duplicate oropharyngeal swab eluates taken daily for 5 days after drug administration.

**Funding:**

Wellcome Trust.

## Introduction

Effective SARS-CoV-2 antivirals taken early in the course of COVID-19 illness accelerate viral clearance, hasten symptom resolution, reduce transmission, and lower the probability of progression to severe disease.^1–4^ Several small molecule drugs and monoclonal antibodies have proven antiviral efficacy in COVID-19, although monoclonal antibodies are no longer used widely, because immune evasion resulting from viral evolution has reduced or abrogated their antiviral effects. The most effective approved small molecule antiviral drug is ritonavir-boosted nirmatrelvir, a main (3C-like) protease inhibitor.^5^ Nirmatrelvir reduced progression to severe disease in an unvaccinated high-risk population by around 90%.^3^ However, the combination drug is expensive and frequently results in troubling dysgeusia, and ritonavir is often contraindicated because of drug–drug interactions.^3^ The development of potentially better tolerated (eg, the main protease inhibitor ensitrelvir^6^) and more affordable drugs that could be administered more widely would be of considerable public health value. To guide policies and practices, the antiviral activities of new drugs need to be evaluated. Antiviral interventions can be assessed and compared using acceleration in the rate of viral clearance as a surrogate for clinical benefit.^7–9^

The natural history of SARS-CoV-2 infection has changed markedly over the 4 years since the beginning of the pandemic.^10^ Serious clinical outcomes—notably, life-threatening inflammatory pneumonitis—are now very rare. As a result, it has become very difficult to demonstrate clinical efficacy for new antiviral drugs, because the required trial sample sizes have become prohibitively large. This difficulty was illustrated in the very large PANORAMIC trial of molnupiravir in the UK, in which only 203 primary events were observed in more than 25 000 randomly assigned at-risk patients.^11^ An alternative approach is to use rates of in-vivo viral clearance to characterise and compare antiviral efficacies,^12^ which is relatively straightforward and requires orders of magnitude fewer patients.^13^ PLATCOV is an ongoing, multicentre, phase 2, adaptive, open-label, randomised, pharmacometric platform trial in symptomatic, low-risk adults with COVID-19 (NCT05041907).^13^ Results from this trial have demonstrated the utility of in-vivo viral clearance in identifying ineffective drugs and assessing and comparing those that are clinically effective.^5,13–16^

Viral clearance in COVID-19 follows an approximate biexponential (biphasic) decay pattern.^17–19^ Effective antiviral interventions increase the rate of viral clearance in the first phase.^12,20^ The effect of antivirals on the second phase is less clear and of lesser importance, because viral densities during the second phase are usually fairly low (ie, unlikely to be transmissible), close to the limit of detection, and clear spontaneously in individuals who are not immunocompromised. Most small molecule drug treatments are given for up to 5 days (eg, remdesivir, molnupiravir, ritonavir-boosted nirmatrelvir) and have short elimination half-lives. Because the primary aim of PLATCOV is to characterise and compare antiviral effects during the first phase of viral clearance, the primary endpoint included measured viral densities only up to day 7. Here, we present an analysis of viral clearance in all patients with unblinded data in the PLATCOV trial, with the aim to characterise temporal changes in viral kinetics and re-assess the optimal approach for characterising and comparing antiviral effects in vivo.

## Methods

### Study design and participants

PLATCOV provides a standardised quantitative comparative method for in-vivo assessment of potential antiviral treatments in early symptomatic COVID-19. Patients were recruited in Thailand, Brazil, Laos, and Pakistan. The primary endpoint is the rate of viral genome clearance estimated under a linear model fitted to the serial log viral densities (measured by quantitative PCR [qPCR] in daily duplicate oropharyngeal viral swab eluates) between days 0 and 7 (8 days in total), denoted α_0−7_. All patients receive symptomatic treatment (mainly paracetamol).

PLATCOV is coordinated and monitored by the Mahidol Oxford Tropical Medicine Research Unit in Bangkok, Thailand. The trial is overseen by a trial steering committee and is conducted according to Good Clinical Practice principles. The trial was approved by the Oxford University Tropical Research Ethics Committee (Oxford, UK) and ethics committees in each country (appendix p 20). The results are reviewed regularly by a data and safety monitoring board.

Adults patients were eligible for the study if they were previously healthy with symptomatic COVID-19 (positive lateral flow test or PCR with a cycle threshold [Ct] value <25), had symptoms for 4 days or less, were at low risk of deterioration (eg, younger than 50 years of age), and gave fully informed consent for full participation in the study (detailed inclusion and exclusion criteria for the PLATCOV trial are provided in the appendix [p 19]).^5,13–16^

Block randomisation (block size of four times the number of concurrent active arms) was performed for each site using a centralised web-based application. The no study drug arm (unblinded, no placebos used) comprised at least 20% of patients at all times, with uniform randomisation ratios applied across the active treatment arms. The laboratory team were masked to treatment allocation and the clinical investigators were masked to the virology results until the study arm was terminated. Apart from the trial statisticians (JAW and PW), the clinical investigators were all masked to the qPCR results.

Patients were included in this analysis if they had been randomly assigned to a currently unblinded treatment arm and had at least 2 days of follow-up (ie, sufficient to estimate a clearance slope). The drugs or monoclonal antibodies evaluated in the PLATCOV trial were ivermectin (until April 11, 2022), remdesivir (until June 10, 2022), casirivimab–imdevimab (Thailand only, until Oct 20, 2022), favipiravir (until Oct 30, 2022), molnupiravir (until Feb 22, 2023), fluoxetine (until May 8, 2023; data not included in this analysis), tixagevimab–cilgavimab (until July 4, 2023; data under analysis), nitazoxanide (Brazil, Laos, and Pakistan, from Jan 18, 2022; ongoing), ensitrelvir (Thailand and Laos only, from March 17, 2023; ongoing), and ritonavir-boosted nirmatrelvir (from June 6, 2022; ongoing as a positive control).

### Procedures

All treatments were observed directly or by video. Oropharyngeal swabs were taken by trained study nurses. A flocked swab (Thermo Fisher MicroTest [Thermo Fisher, Waltham, MA, USA] and later COPAN FLOQSwabs [COPAN Diagnostics, Murrieta, CA, USA]) was rotated against the tonsil 360° four times and placed in Thermo Fisher M4RT (Thermo Fisher, Waltham, MA, USA) viral transport medium (3 mL).^5,13–16^ Swabs were transferred at 4–8°C, aliquoted, and then frozen at –80°C within 48 h.

On day 0, following randomisation, four separate swabs (two from each tonsil) were taken. Separate swabs from each tonsil were then taken once daily from day 1 to day 7, on day 10, and on day 14 (ie, 22 swabs). Each swab was processed and tested separately. Vital signs were recorded three times daily by the patient (initial vital signs on day 1 were recorded by the study team), and symptoms and any adverse effects were recorded daily in a case report form. The TaqCheck SARS-CoV-2 Fast PCR Assay (Applied Biosystems, Thermo Fisher Scientific, Waltham, MA, USA) quantitated viral density (RNA copies per mL). This multiplexed real-time PCR method detects the SARS-CoV-2 *N* and *S* genes and human *RNase P* gene in a single reaction. *RNase P* is used to adjust for variation in intracellular viral RNA. Whole-genome sequencing was performed to identify viral variants (appendix pp 2–4).

### Statistical analysis

Oropharyngeal eluate viral densities were quantified by PCR on 96-well plates. Each plate contained ten or 12 ATCC controls (Manassas, VA, USA; these are heat-inactivated SARS-CoV-2 viruses [VR-1986HK strain 2019-nCoV/USAWA1/2020]) varying from 10 to 10^6^ copies per mL. We fitted a linear mixed-effects model to all ATCC control data from all available plates (using R package lme4 version 1.1.34), with the genome copies per mL on the log_10_ scale (ie, a linear relationship between Ct values and known log_10_ genomes per mL). The model included fixed effects on the slope and intercept by laboratory (reference laboratory was Thailand) and random effects on the slope and intercept by plate. Visual checks were done to ensure that controls were in a reasonable range. The mixed-effects model was then used to transform the observed Ct values into log_10_ genomes per mL. A Ct value of 40 was considered left-censored, and the plate-specific censoring value was used in subsequent analyses (appendix p 7).

The baseline viral density was defined as the geometric mean SARS-CoV-2 density in the oropharyngeal eluates of the four swabs taken before randomisation. Temporal trends in baseline viral densities were characterised using generalised additive models with penalised splines, as implemented in the mgcv package version 1.9.0. Because the timing of patient recruitment relative to symptom onset could also have changed over time (and this could affect baseline viral densities), the temporal effect was stratified by the reported interval since symptom onset (1 day, 2 days, 3 days, or 4 days). Pearson correlation coefficients between baseline covariates were estimated using the R function cor.test.

The analysis of the serial viral density data used the same core analytical model as reported previously.^5,13–16^ We characterised oropharyngeal viral clearance under a single exponential decay model (linear decay on the log scale). Under this model, the rate of viral clearance is defined as the slope parameter of the linear fit (model likelihood given in the appendix [p 5]). Covariate terms for the slope and intercept were the reported days since symptom onset, study site, age, sex, and number of vaccine doses received. This model parameterised the treatment effect relative to a reference intervention (eg, no study drug) as a proportional change $\left(e^{b_{T(i)}}\right)$. As a sensitivity analysis, we parameterised the treatment effect as an additive change $\left(b_{0}e^{b_{i}+b_{cov}}+b_{T(i)}\right)$. Model comparison was done using leave-one-out as implemented in the package loo version 2.6.0.

All models were fitted using weakly informative priors on all parameters (appendix pp 5–6). These priors help computational convergence but have no effect on the parameter estimates.^13^ Previously, we also used a non-linear up-down model (linear increase followed by linear decrease), but this also had no effect on treatment effect estimates.^13^

To assess the temporal changes in viral clearance, we added a penalised B-spline of degree 4 to the population mean intercept *a*_0_ (baseline viral density) and population mean slope *b*_0_ (population viral clearance rate) in the reference group (for most analyses, this is the no study drug arm). This was done by having many knots at regular intervals (20 knots in the main analysis), with an informative penalisation prior on parameter changes across knots. The penalisation prior governs the smoothness of the spline fit.

The viral clearance half-life of viral clearance for individual *i*, denoted $t_{1/2}^{i}$ is defined as

$$t_{1/2}^{i}=\log_{10}(0\cdot 5)/{\text{slope}}_{i}.$$

The population viral clearance half-life for each arm is defined as

$$t_{1/2}^{T(i)}=\log_{10}(0\cdot 5)/\left(b_{0}{\text{e}}^{bT(i)}\right),$$

where *b*_0_ is the population viral clearance rate, and e^bT(*i*)^ is the treatment effect.

In the meta-analysis, we adjusted for temporal confounding by explicitly incorporating into the model the temporal change in the population mean clearance rate. We fitted the full Bayesian linear model with a spline term on the clearance rate in the no study drug arm (which spans the entire study period) as a function of the calendar date. As a sensitivity analysis, we assessed treatment effect heterogeneity with respect to the SARS-CoV-2 major lineages. This was done by incorporating interaction terms between the intervention and the viral lineage.

Given the potential changes in the viral clearance kinetics of SARS-CoV-2 over the pandemic, we used the available comparative data to assess whether the current trial design is optimal for pharmacometric assessment and to determine the optimal design that would facilitate the rapid identification and evaluation of effective antivirals. We defined optimal as the design (duration and frequency of sampling) that maximised the expected Z score for differences in viral clearance rates when comparing an effective randomised intervention with the concurrent no treatment arm or comparing two concurrently randomised interventions with different antiviral efficacies. The Z score is the estimated effect size divided by the estimated standard error. We bootstrapped the data (sampling patients in each comparison with replacement) to obtain uncertainty intervals for the Z score estimates for each comparison. For the Z scores to be comparable, each bootstrap sample contained 50 patients per group. Three designs were compared: (1) varying the duration of follow-up from 2 days (ie, using qPCR measurements taken on days 0, 1, and 2) to 14 days (using all available qPCR data); (2) varying the number of swabs taken each day (one or two); and (3) comparing twice daily swabs taken on days 0–4 (ten qPCR measurements), every other day (days 0, 2, and 4; six qPCR measurements), or only on days 0 and 4 (four qPCR measurements). Empirical expected Z scores were estimated under the linear model for five separate intervention comparisons (each comparison used concurrently randomised patients): remdesivir versus no study drug, casirivimab–imdevimab versus no study drug, molnupiravir versus no study drug, ritonavir-boosted nirmatrelvir versus no study drug, and ritonavir-boosted nirmatrelvir versus molnupiravir. The data for ritonavir-boosted nirmatrelvir versus no study drug spanned 16 months with a brief hiatus in recruitment from January to February, 2023, so we arbitrarily split these data into two separate comparisons to make a total of six comparisons: before January, 2023, and after February, 2023. This split allowed assessment of how much the temporal change in viral clearance was driving the observed results. For each of these six comparisons, and each sampling design (duration of follow-up and number of samples), we bootstrapped the data 50 times (sampling the patients with replacement) and fitted the linear model to estimate the treatment effect and standard error.

As a sensitivity analysis, we also conducted a meta-analysis to estimate the treatment effects using serial oropharyngeal swabs, using data up to the optimal follow-up duration which maximise the Z scores. We then compared the estimated treatment effects to those obtained using the current follow-up duration of 7 days.

All Bayesian models were written in stan and fitted using the rstan interface version 2.32.3. All analyses were done using R version 4.3.2.

PLATCOV is registered at ClinicalTrials.gov, NCT05041907.

### Role of the funding source

The funder of the study had no role in study design, data collection, data analysis, data interpretation, or writing of the report.

## Results

**Figure 1:**
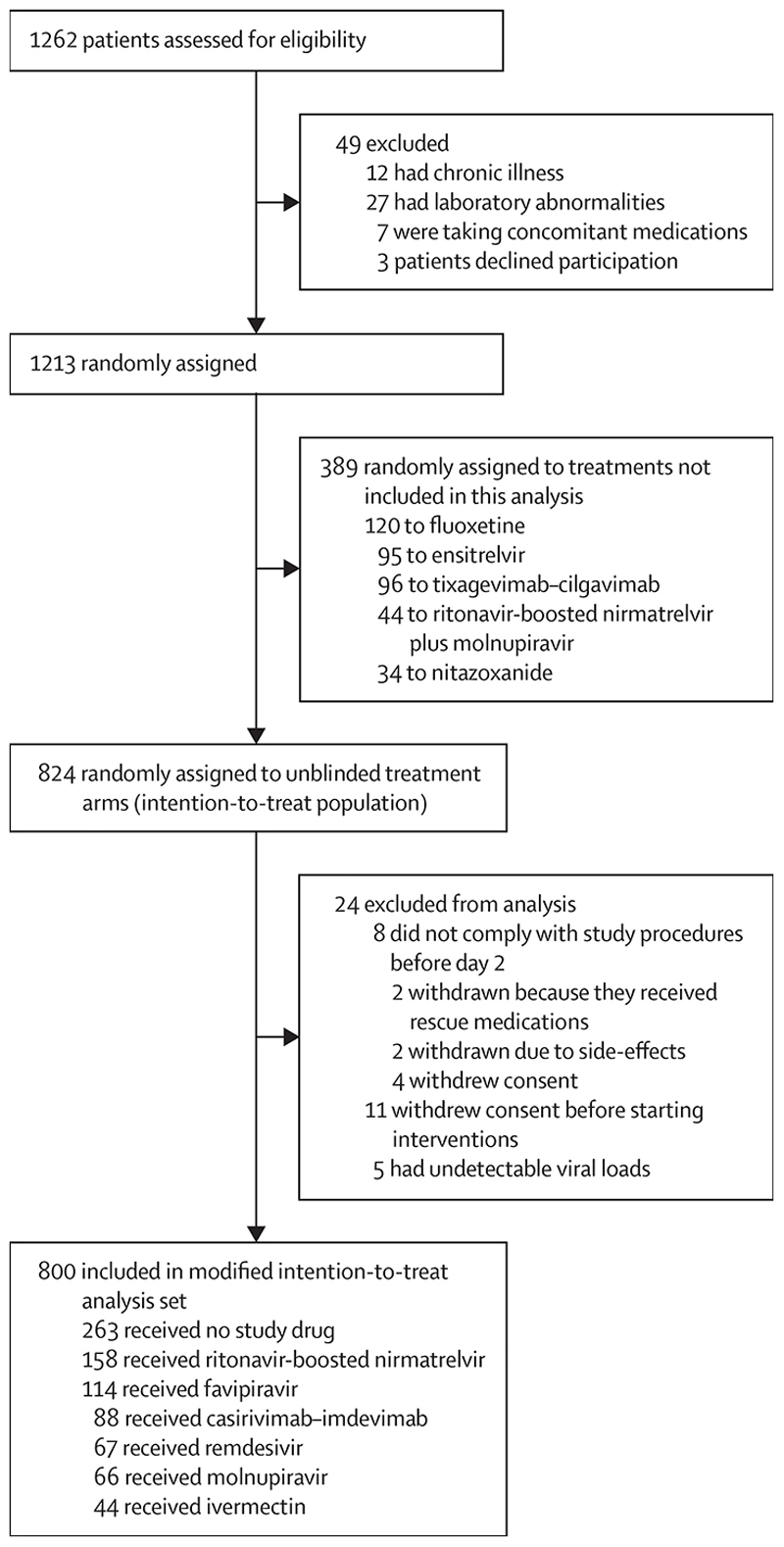
PLATCOV trial profile and selection of patients for this analysis This analysis included patients enrolled between Sept 30, 2021, and Oct 20, 2023, who met the modified intention-to-treat criteria and whose viral clearance data have been unblinded and published. Patients were excluded from the modified intention-to-treat population if protocol deviations occured on days 0–2.

Between the Sept 29, 2021, and Oct 20, 2023, 1262 patients across six sites in four countries (Thailand, Brazil, Pakistan, and Laos) were randomly assigned in the PLATCOV trial. After excluding patients who withdrew consent, who were not SARS-CoV-2 positive on any follow-up samples, or who had fewer than 2 days follow-up, the analysis population consisted of 800 patients randomly assigned across seven arms (not all con-currently; appendix p 8; figure 1). 783 (98%) of 800 patients had received at least one vaccine dose, and 703 (88%) were fully vaccinated before symptom onset (table; appendix p 9). 714 (89%) patients were recruited at one site in Thailand (Hospital for Tropical Diseases, Bangkok). The mean interval from symptom onset to randomisation was 2·1 days (SD 0·8), and the geometric mean baseline viral density in oropharyngeal eluates was 5·5 log_10_ genomes per mL (1·2). 780 (98%) patients had complete viral density data between days 0 and 7 (appendix p 10).

**Figure 2:**
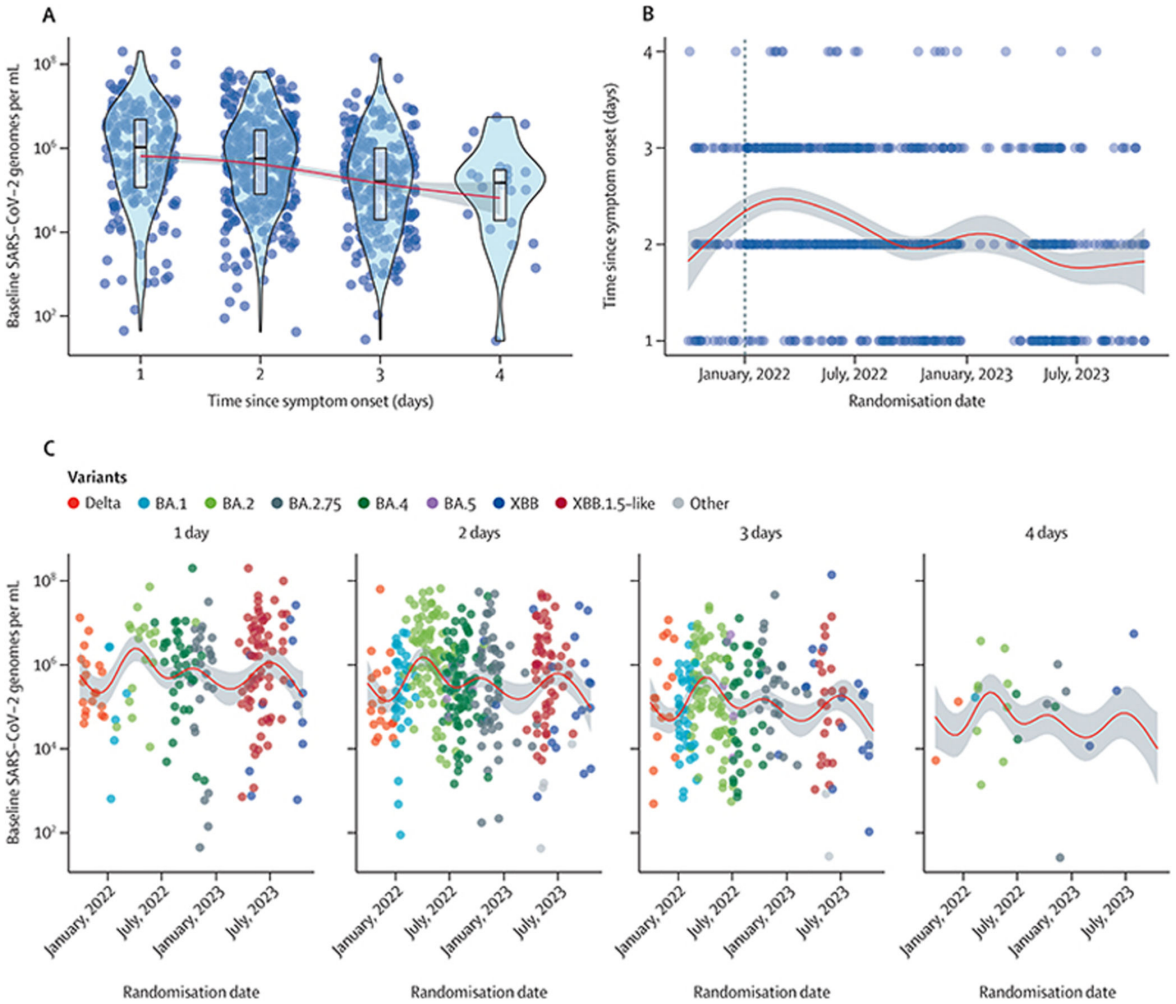
Changes in symptoms duration at enrolment and baseline oropharyngeal eluate viral densities over a 2-year period (2021–23) (A) The association between reported interval since symptom onset and baseline viral density. (B) Temporal changes in the reported interval since symptom onset. The vertical dashed line indicates the first omicron BA.1 infection enrolled in the study. (C) Temporal changes in the baseline viral density stratified by reported interval since symptom onset. Red lines represent mean estimated values and shaded areas the 95% CIs under a generalised additive model.

The baseline oropharyngeal eluate viral densities remained high over the 2-year period (appendix p 11), but there were systematic trends over time associated with different SARS-CoV-2 variants. The reported interval since symptom onset was negatively correlated with the baseline viral density (ρ=–0·22, 95% CI –0·29 to –0·16; *R*^2^ 0·05; figure 2A). Each reported additional day since symptom onset corresponded to a 1·9-times (95% credible interval [Crl] 1·5 to 2·4) decrease in the baseline viral density, and men had 1·4-times (95% Crl 1·0 to 2·1) higher baseline viral load densities than women (appendix p 12). There were small changes in the mean reported number of days since symptom onset over time. For example, during the omicron BA.1 wave (Jan 1–March 11, 2022) patients were recruited slightly later on average (figure 2B). In a multivariable spline model stratified by the interval since symptom onset, there was evidence of systematic temporal changes in baseline viral density over time that were not explained by differences in time from symptom onset (figure 2C). Because these are observational data, causality cannot be determined (eg, whether these differences result from variant-specific mutations in the spike protein), but the data are compatible with higher peak viral densities with specific variants such as BA.2 and XBB.1.5-like.

**Figure 3:**
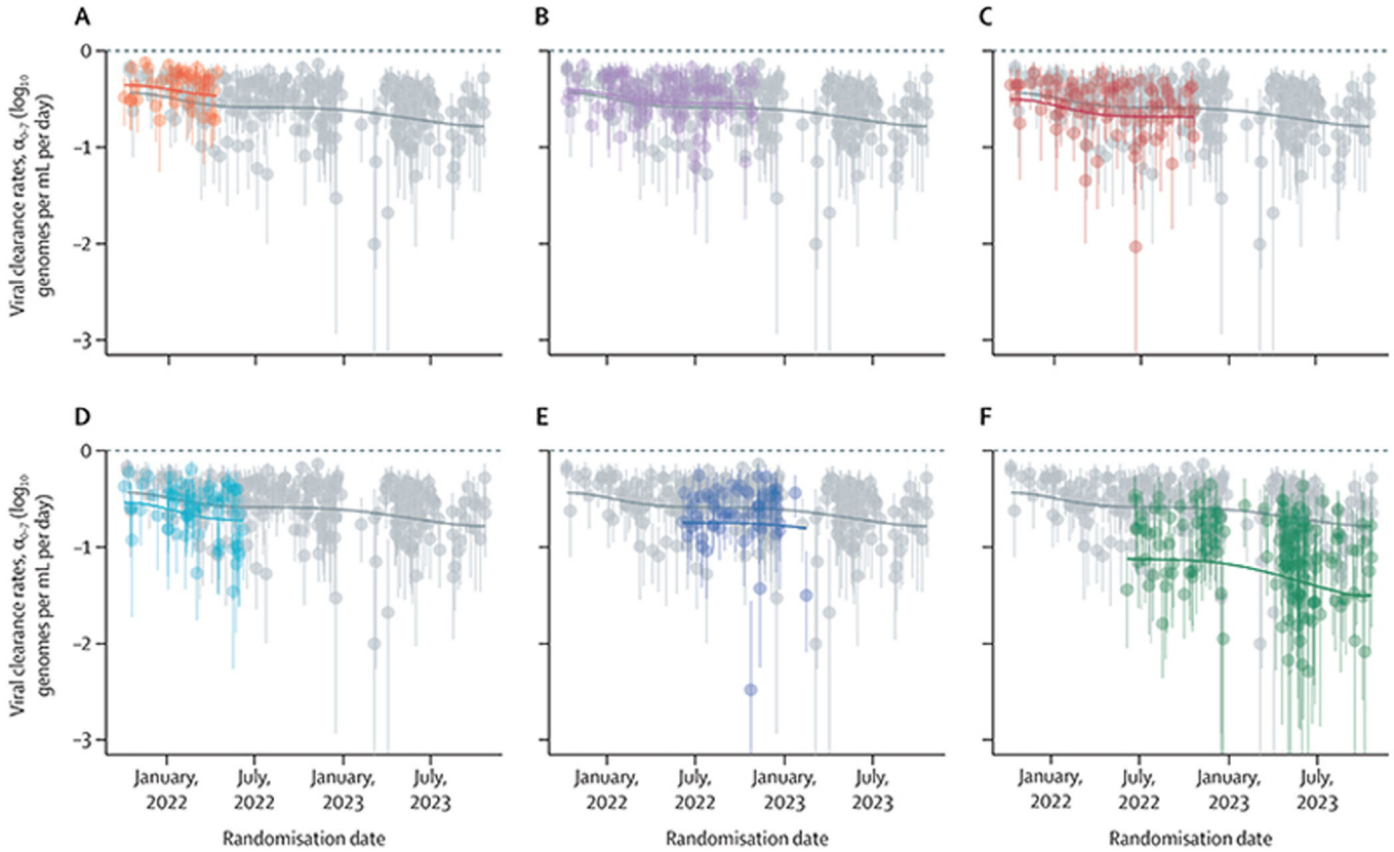
Individual patient data meta-analysis showing change over time in estimated rates of viral clearance between days 0 and 7 (α_0−7_) (A) Ivermectin. (B) Favipiravir. (C) Casirivimab–imdevimab. (D) Remdesivir. (E) Molnupiravir. (F) Ritonavir-boosted nirmatrelvir. Average clearance rates for each intervention (coloured lines) and the no study drug arm (grey line) were estimated from a spline fit. Treatment effects were parameterised as a proportional change in rate. The grey circles and grey lines for the no study drug arm are identical in each panel. Vertical lines show 95% credible intervals under the linear model. A negative sign of the clearance rate indicates a decreasing directional change in viral density. α_0−7_=the rate of viral genome clearance estimated under a linear model fitted to the serial log viral densities (measured by qPCR in daily duplicate oropharyngeal viral swab eluates) between days 0 and 7.

Viral clearance increased substantially over 2 years of the pandemic, as shown clearly in patients assigned to no study drug (appendix p 13). Figure 3 shows the individual clearance rate estimates, α_0−7_, with a spline term to highlight temporal changes. In the no study drug arm, median viral clearance rates doubled from –0·43 log_10_ genomes per mL per day in September, 2021 (corresponding to a half-life of 16·6 h [IQR 15·3 to 18·2]), to –0·78 log_10_ genomes per mL per day in October, 2023 (half-life 9·2 h [8·0 to 10·6]). This change corresponds to a relative shortening in viral clearance half-life of 44% (95% CrI 19 to 64) over 2 years. Similar trends were noted for the treated individuals (appendix p 17). For example, the mean viral clearance rate in the ritonavir-boosted nirmatrelvir arm increased from –1·12 log_10_ units per day in June, 2022 (half-life 6·4 h [IQR 5·7 to 7·3]), to –1·50 log_10_ units per day in October, 2023 (half-life 4·8 h [4·2 to 5·5]). This corresponds to a relative shortening in viral clearance half-life of 26% (95% CrI –4 to 42). The reduction of viral clearance half-life was most apparent early in the study between September, 2021 (delta variant), and mid-February, 2022 (BA.2 variant; appendix p 14). Subsequently, the half-life plateaued at 12·3 h (95% CrI 8·4 to 17·0 h) in July, 2022, during the BA.2, BA.4, BA.5, and BA.2.75 variants, and gradually reduced again after the emergence of XBB and XBB.1.5-like variants in January, 2023. There was no clear relationship between individual viral clearance rate estimates and the number of days since symptom onset, sex, age, or the number of vaccine doses received (appendix p 12).

**Figure 4:**
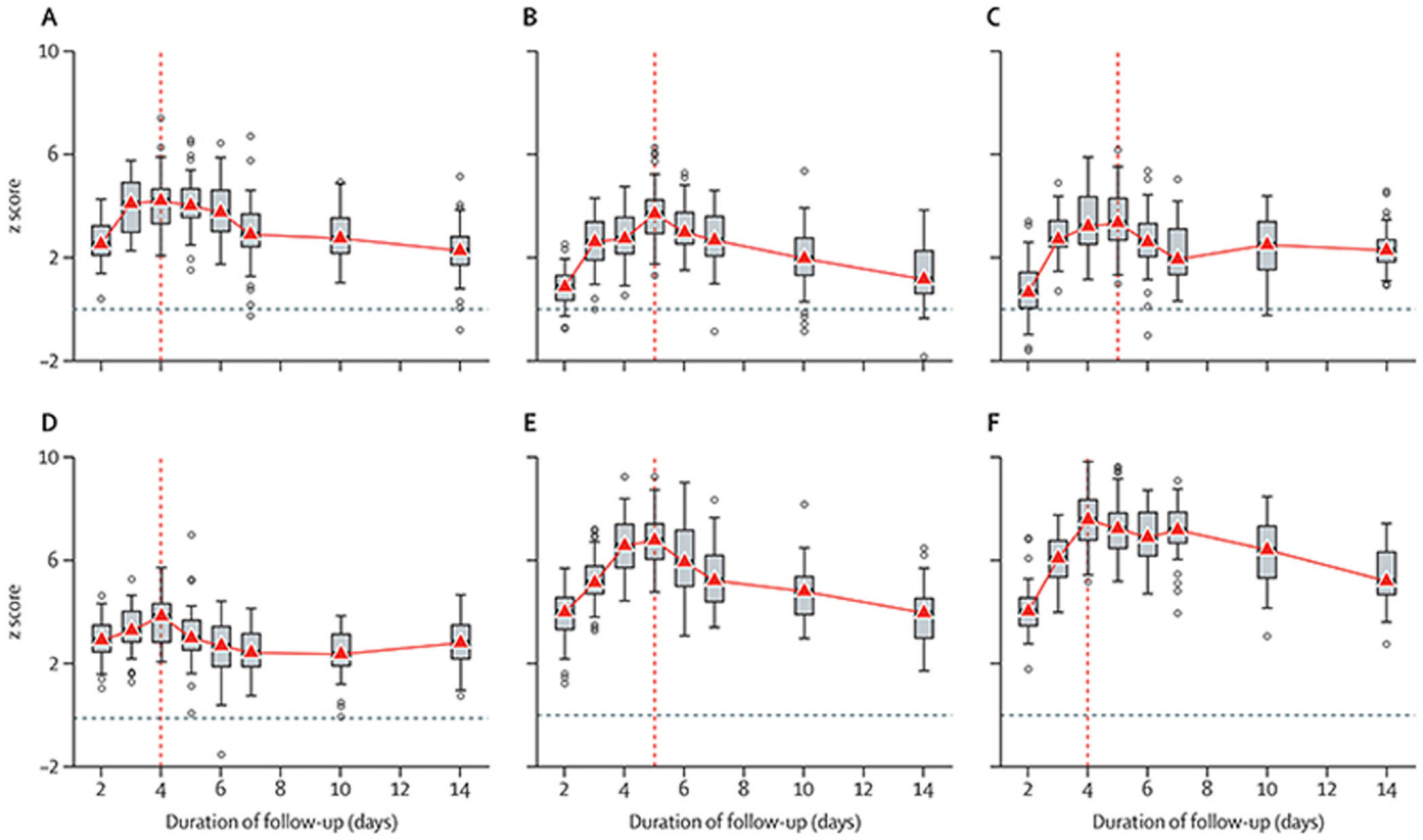
Z scores for the six comparisons of treatment effect as a function of the follow-up duration (A) Remdesivir versus no study drug. (B) Molnupiravir versus no study drug. (C) Casirivimab–imdevimab versus no study drug. (D) Nirmatrelvir versus molnupiravir. (E) Nirmatrelvir versus no study drug (before February, 2023). (F) Nirmatrelvir versus no study drug (after February, 2023). Boxplots show the median IQR of the Z scores for 50 bootstrap iterations. Each bootstrap dataset contained 50 patients per group. Vertical dashed lines indicate the follow-up durations with maximal Z scores. All comparisons use concurrent controls only.

Figure 4 shows the expected Z scores for six randomised comparisons with sample sizes of 50 patients per group as a function of the duration of data included in the estimated viral clearance rate (varying from days 0–2 to days 0–14). For all pairwise comparisons there was a clear inverted-parabolic relationship between the expected Z score and the duration of follow-up. The expected Z score was maximised for durations between 4 days and 5 days, implying that 4–5 days follow-up is optimal in terms of statistical power when the data are analysed under a linear model framework. Fitting a single component log-linear model over a longer period systematically reduced the slope estimate (ie, lengthened the half-life), because it incorporated more of the slower β-phase (second phase) of viral elimination in the estimate. Whereas, reducing the frequency of the viral density measurements from twice daily on days 0–4, to every other day, and to only on days 0 and 4 reduced the expected Z score but with a lesser effect than reducing the number of swabs taken on each day from two to one (appendix p 15).

**Figure 5:**
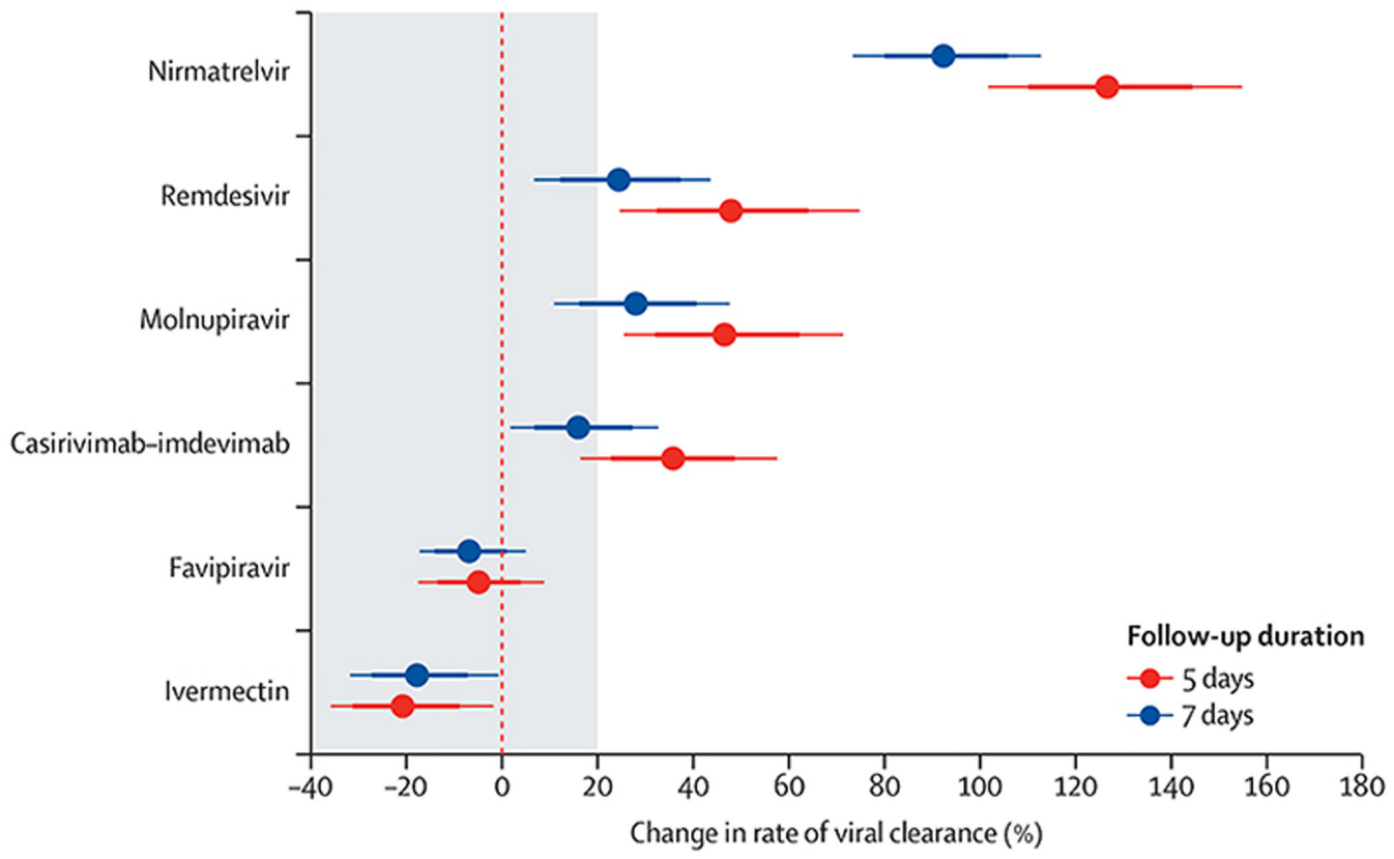
Individual patient data meta-analysis of the treatment effect of the six randomised interventions relative to no study drug The models were adjusted for temporal changes in viral clearance in the no study drug arm using penalised B-splines. Points represent the median posterior estimate and thick and thin lines the 80% and 95% credible intervals, respectively.

Adjusting for temporal changes in clearance rates under the linear model, there was a clear hierarchy between the studied interventions (figure 5). This hierarchy remained consistent when estimating treatment effects using the average viral clearance rates until day 5 (α_0−5_) or until day 7 (α_0−7_). Ritonavir-boosted nirmatrelvir had the greatest effect (appendix p 18). The small molecule drugs remdesivir and molnupiravir had similar effects to each other (appendix p 18). The average treatment effect for the monoclonal antibody casirivimab–imdevimab was of similar magnitude to molnupiravir (ignoring known treatment effect heterogeneity;^14^ appendix p 18). This meta-analysis confirmed the absence of any measurable effect of high-dose ivermectin or high-dose favipiravir. For all four effective interventions, the analysis using the α_0−5_ average clearance rates estimated substantially larger effect sizes, albeit with slightly wider uncertainty intervals, than the analysis using the α_0−7_ average clearance rates. There was no evidence of treatment effect heterogeneity for the small molecule drugs by viral variants, whereas the effect of casirivimab–imdevimab varied considerably across the major viral variants (appendix p 16).

## Discussion

SARS-CoV-2 oropharyngeal clearance rates in uncomplicated SARS-CoV-2 infections substantially increased over 2 years of the pandemic. Natural viral clearance in October, 2023, was twice as fast as it was in September, 2021. This granular, prospectively gathered dataset confirms the findings of other larger scale observational cohorts.^21^ In this studied cohort, in which most participants were fully vaccinated, waves of different viral variants succeeded each other, following a generally similar pattern to that observed in most areas of the world. There was no clear association between particular viral variants and increases in viral clearance rates. Instead, there appears to have been a steady increase in clearance rates across all variants over time. Some variants (eg, BA.2.75) were clearly associated with higher baseline viral densities, which was not explained by differences in the interval from symptom onset. It is not possible to ascribe with confidence the underlying cause for these higher baseline viral loads, but it would be compatible with either differences in viral replication^22^ or differences in tropism or immunity.^23^

The substantial acceleration in natural viral clearance over 2 years of the pandemic presumably reflects the interplay between the acquisition of immunity and the antigenic changes in the evolving variants. This acceleration has implications for the assessment of in-vivo antiviral activity. SARS-CoV-2 oropharyngeal or nasopharyngeal clearance is biphasic.^17–19^ Effective drugs substantially accelerate the first phase. In September, 2021, when viral clearance rates were much slower, the inflexion in the clearance curve (transition from the first to the second slower phase) was close to 7 days, so fitting a single rate constant to the log-linear decline in viral densities over 7 days incurred relatively little bias. However, the interval from presentation to the inflexion point has shortened progressively, so forcing a single rate constant to the serial qPCR values over 7 days now incurs greater bias, resulting in progressive underestimation of the initial phase rate of clearance. This is important for historical comparisons of antiviral activity because, with any viral clearance measure, drugs tested more recently will result in faster viral clearance than they did earlier in the pandemic. Moderately effective drugs evaluated earlier in the pandemic (eg, remdesivir) resulted in viral clearance rates that were similar to those in the no treatment arm of the study later in the pandemic.

The PLATCOV study characterised the effects of several antiviral drugs on viral clearance,^5,13–16,24^ with findings that were generally consistent with earlier clinical trials assessing their efficacy in the prevention of disease progression. Comparative estimates of in-vivo antiviral activity allow for rational selection of drugs now that comparisons based on clinical endpoints are no longer feasible because of the prohibitively large sample sizes required in clinical trials. In this analysis, using the observed differences in the viral clearance profiles between effective and ineffective drugs or the no treatment arm allowed determination of the sampling duration that best characterised these differences. The greatest differences between effective and ineffective (or no) drugs were observed for assessments made over 4–5 days. Although there was substantial inter-individual variation in clearance rates, and intra-individual variation between the serial viral density estimates, with current viral clearance rates daily sampling still has adequate discriminatory power.

However, if natural viral clearance continues to accelerate, then it might be necessary to sample twice daily over a shorter period. Shortening the viral clearance serial sampling to 5 days simplifies the comparative assessment of antiviral drugs in COVID-19 (although later sampling is still necessary if rebound is being assessed).

These data emphasise the importance of fixed ratio (as opposed to response adaptive randomisation ratios) randomisation and contemporary comparators in COVID-19 platform trials. Temporal confounding across non-concurrently randomised interventions or for time-varying randomisation ratios (this occurs in response-adaptive trials) requires model-dependent adjustment. Even an ineffective drug will appear effective if compared with a historical control. The exact ranking of all unblinded interventions in the PLATCOV platform trial in the meta-analysis is dependent in part on correct adjustment for the temporal trends. This issue is particularly salient for the comparison between remdesivir and molnupiravir, because they had very similar effects, and it can be difficult to differentiate small effects from small biases.

Today, SARS-CoV-2 is predominantly a mild infection in vaccinated individuals and does not require specific antiviral treatment, justifying the recruitment of patients into the pharmacometric assessment who receive no specific treatment. However, in patients with underlying conditions, or older patients, COVID-19 is potentially dangerous, and specific antiviral treatment is required. There is no reason to believe that relative antiviral activities are different in these high-risk subgroups to those observed in low-risk patients. At the beginning of the pandemic there were no effective interventions, so identification of minor accelerations in viral clearance was relevant. Today, modest acceleration in the rate of viral clearance might still be relevant for chemo-prevention,^24^ but it is very unlikely that drugs that are less effective than those currently being used would be deployed for the treatment of symptomatic COVID-19. The simple methodology of the PLATCOV trial is well tolerated in symptomatic adults and is efficient, because it identifies efficacious antivirals (ie, those that accelerate viral clearance rates >20% compared with no drug) with sample sizes that are usually fewer than 40 studied patients per group.

Although this is the largest detailed pharmacometric study in COVID-19, it has some limitations. Most of the patients were enrolled in Bangkok, Thailand, so the temporal trends observed could be different in other parts of the world. The causes of the substantial inter-patient variations in viral clearance and the overall acceleration in viral clearance over 2 years of the pandemic have not been characterised adequately. More than 95% of patients were vaccinated before the enrolment infection, so we could not characterise differences in treatment effects between vaccinated and unvaccinated individuals. Although there is a clear rationale for using viral clearance as a surrogate endpoint in assessing therapeutics,^8,9^ additional data are still needed to characterise the association between acceleration in viral clearance and clinical outcomes, such as rate of symptom clearance.

In summary, SARS-CoV-2 viral clearance accelerated substantially over 2 years of the pandemic, necessitating a shortening of the sampling time to evaluate and compare antiviral drugs efficiently.

## Supplementary Material

Appendix

## Figures and Tables

**Table:** Baseline demographics

	No study drug (n=263)	Ritonavir-boosted nirmatrelvir (n=158)	Favipiravir (n=114)	Casirivimab-imdevimab (n=88)	Remdesivir (n=67)	Molnupiravir (n=66)	Ivermectin (n=44)
Country of enrolment							
Brazil	26 (10%)	4 (3%)	16 (14%)	0	9 (13%)	0	0
Thailand	230 (88%)	150 (95%)	98 (86%)	88 (100%)	58 (87%)	65 (99%)	44 (100%)
Laos	3 (1%)	4 (3%)	0	0	0	1 (2%)	0
Pakistan	4 (2%)	0	0	0	0	0	0
Age, years	31·1 (8·1)	31·3 (8·9)	30·2 (7·5)	27·9 (7·3)	30·1 (8·2)	31·3 (7·5)	30·0 (7·0)
Sex							
Female	173 (66%)	109 (69%)	71 (62%)	55 (62%)	35 (52%)	37 (56%)	24 (55%)
Male	90 (34%)	49 (31%)	43 (38%)	33 (38%)	32 (48%)	29 (44%)	20 (34%)
Weight, kg	63·0 (13·6)	61·5 (12·3)	63·0 (13·6)	60·4 (12·3)	63·9 (11.0)	63.4 (14·7)	61·6 (12·3)
BMI, kg/m^2^	23·2 (4.1)	23·0 (3·8)	23·1 (3·7)	22.1 (3.1)	22.7 (3.1)	23·1 (4·0)	22.3 (3.2)
Interval since symptom onset, days	2 (1–3)	2 (1–2)	2 (2–3)	2 (2–3)	2·5 (2–3)	2 (2–2)	2 (2–3)
Admission viral load, log_10_ genomes per mL	5.6 (4·7–6·4)	5·6 (4·6–6·4)	5·5 (4·7–6·2)	5.7 (5·0–6·4)	5.3 (4·8–6·3)	5·8 (5·0–6·4)	5·6 (5·0–6·6)
Vaccinated	253 (96%)	153 (97%)	112 (98%)	85 (97%)	64 (96%)	65 (99%)	43 (98%)
Start date	Sept 29,2021	June 5, 2022	Oct 10, 2021	Oct 1, 2021	Oct 4, 2021	June 5, 2022	Sept 29,2021
Finish date	Oct 20, 2023 (ongoing)	Oct 20, 2023 (ongoing)	Oct 29, 2022	Oct 19, 2022	June 7, 2022	Feb 14, 2022	April 11, 2022
SARS-CoV-2 variant							
Delta	10 (4%)	0	11 (10%)	13 (15%)	10 (15%)	0	12 (27%)
BA.1	13 (5%)	0	21 (18%)	15 (17%)	20 (30%)	0	14 (32%)
BA.2	52 (20%)	1 (1%)	42 (37%)	30 (34%)	37 (55%)	5 (8%)	18 (41%)
BA.2.75	43 (16%)	30 (19%)	5 (5%)	5 (6%)	0	28 (42%)	0
BA.4	2 (1%)	3 (2%)	3 (3%)	0	0	2 (3%)	0
BA.5	42 (16%)	26 (17%)	32 (28%)	25 (28%)	0	28 (42%)	0
XBB	29 (11%)	22 (14%)	0	0	0	3 (5%)	0
XBB.1.5-like	67 (26%)	75 (48%)	0	0	0	0	0
Other	5 (2%)	1 (2%)	0	0	0	0	0

Data are n (%), mean (SD), or median (IQR).

## Data Availability

All data and code necessary to reproduce the results in this analysis are openly available on GitHub. All code and de-identified participant data required for replication of the study’s endpoints are openly accessible through Zenodo, as well as the study protocol and statistical analysis plan, from publication date onwards. Individual patient data can be requested and may be shared according to the terms defined in the Mahidol Oxford Tropical Medicine Research Unit data sharing policy with other researchers to use in the future from the date of publication. Further information on how to apply is on the Mahidol Oxford Tropical Medicine Research Unit Tropical Health Network website.

## Research in context

### Evidence before this study

Antiviral interventions can be assessed and compared using acceleration in the rate of viral clearance as a surrogate for clinical benefit. We searched PubMed for studies published in English from Jan 1, 2020, to Jan 3, 2024, using the terms: (SARS-CoV-2) AND (randomised) AND (antiviral) AND (viral clearance OR viral kinetics). Most phase 2 studies evaluated viral densities in clinical samples as primary endpoints, with variation in both the number of sampling timepoints and follow-up duration. Most studies quantified viral clearance as the time to PCR negativity or the difference in viral loads at follow-up relative to the baseline viral density, referred to as least squares mean changes. Few studies have quantified rates of viral clearance or have explicitly considered the factors that might affect the estimated measures of antiviral activity (eg, calendar time, sampling duration, viral variant, and host effects).

### Added value of this study

In a pooled individual patient data meta-analysis of unblinded treatment arms in the PLATCOV platform trial, we show that the clearance rates of SARS-CoV-2 measured in oropharyngeal eluates accelerated substantially over 2 years of the COVID-19 pandemic. In untreated patients, rates of viral clearance were twice as fast in October, 2023, as they were in September, 2021. This has practical implications for the comparative assessment of antiviral drugs. First, the change in viral clearance rates over time can introduce temporal bias when making historical comparisons of antiviral activity. The viral clearance rate following effective treatment in the early phase of the pandemic could have been as rapid as that of untreated individuals in recent infections. Second, we show that only 4–5 days follow-up is necessary to compare antiviral interventions accurately. We propose an improved study design for pharmacodynamic assessment of antivirals for COVID-19.

### Implications of all the available evidence

SARS-CoV-2 viral clearance accelerated substantially over 2 years of the pandemic, resulting in a shortening of the sampling time required for its measurement. At time of writing (October, 2023), a 5-day follow-up duration (as compared to 8–10 days at the start of the pandemic) maximises statistical power when comparing antiviral drugs. Concurrent randomisation and statistical adjustments for temporal trends are necessary to reduce biases when comparing antiviral treatment effects.
