# Supplementary material for: Temporal changes in SARS-CoV-2 clearance kinetics and the optimal design of antiviral pharmacodynamic studies: an individual patient data meta-analysis of a randomised, controlled, adaptive platform study (PLATCOV)
Source: Lancet Infect Dis. Author manuscript; Available in PMC 2025 Jun 11. (PMC7617756; doi:10.1016/S1473-3099(24)00183-X)
Supplement: Appendix [file EMS206165-supplement-Appendix.pdf]

THE LANCET
Infectious Diseases

## Supplementary appendix

This appendix formed part of the original submission and has been peer reviewed. We post it as supplied by the authors.

Supplement to: Wongnak P, Schilling WHK, Jittamala P, et al. Temporal changes in SARS-CoV-2 clearance kinetics and the optimal design of antiviral pharmacodynamic studies: an individual patient data meta-analysis of a randomised, controlled, adaptive platform study (PLATCOV). *Lancet Infect Dis* 2024; published online April 24. https://doi.org/10.1016/S1473-3099(24)00183-X.

# Appendix

## Temporal changes in SARS-CoV-2 clearance kinetics and the optimal design of antiviral pharmacodynamic studies: an individual patient data meta-analysis of a randomised, controlled, adaptive platform study (PLATCOV)

S1: Virus variant determination.................................................. 2
S2: Bayesian hierarchical model.................................................. 5
S3: Supplementary figures.................................................. 7
S4: Supplementary tables.................................................. 17
S5: Inclusion-exclusion criteria.................................................. 19
S6: Ethical approvals.................................................. 20
S7: List of Sites and Investigators (PLATCOV Collaborative Group).................................................. 21
S8: Master protocol of the PLATCOV trial.................................................. 25

# S1: Virus variant determination

## Brazil site – Virus variant determination

***SARS-CoV-2 whole-genome sequencing***

The virus sequencing was carried out using two different technologies, Illumina (Illumina, USA) and IonTorrent (ThermoFisher Scientific, USA). Only SARS-CoV-2-positive samples with Ct < 30 values for virus targets were considered. Illumina libraries were prepared using the QIAseq FX DNA Library Prep kit® (QIAGEN, Germany) and sequenced on the Illumina MiSeq® platform (Illumina, USA) with a v3 (600 cycles) cartridge giving 300bp paired-end reads, following all the manufacturer's protocols. IonTorrent libraries were prepared using the Ion AmpliSeq SARS-CoV-2 Panel® (ThermoFisher Scientific, USA) to give 125 bp to 275 bp amplicons and sequenced single-end on the IonTorrent PGM platform® with a 314-chip kit (ThermoFisher Scientific, USA), according to the manufacturer's recommendations. Three negative controls were used in all sample processing steps (cDNA synthesis, viral genome amplification, and library preparation).

***Viral genome assembly and classifications***

A custom pipeline was used to process the sequencing data. In the first step, quality control was performed with Trimmomatic v0.39. Adapter and primer sequences, short reads (< 50 nucleotides), and low-quality bases (Phred score < 30) were removed. Next, reads were mapped against the SARS-CoV-2 reference genome (GenBank accession: NC_045512) with Bowtie2. Samtools manipulated the mapping files, whilst consensus genome sequences were estimated using the bcftools consensus option. Masking of low-coverage sites was performed with bedtools. The code for the described pipeline can be found on GitHub (https://github.com/filiperomero2/ViralUnity). Depth thresholds differed between

sequencing technologies employed. For IonTorrent data, sites with less than 20-fold depth were masked, while for Illumina, the minimum threshold was 10-fold. Sequences <70% genome coverage breadth were removed from downstream analysis.

## Thailand and Laos sites – Virus variant determination

### *SARS-CoV-2 whole-genome sequencing*

The sequencing method carried out in this experiment follows the "PCR tiling of SARS-CoV-2 virus with rapid barcoding and Midnight RT PCR Expansion" provided by Oxford Nanopore Technology (Oxford, UK) developed based on a protocol by the ARTIC network group[1] and Freed *et al.*[2] Library preparation process started with reverse transcription, which consists of mixing the purified viral RNA with LunaScript RT SuperMix and incubating the mixtures in a thermal cycler. DNA fragments used in the assembly process were amplified by PCR using Midnight primer set (V3) giving 1200bp amplicons and attached with barcodes from Rapid Barcode Plate (RB96). The mixtures from each sample were pooled together, cleaned with AMPure XP Beads (AXP), and attached with Rapid Adapter F (RAP F). The prepared DNA fragments were then loaded into a primed flow cell (FLO-MIN106) and sequenced on GridION MK1 system (MinION Mk1B system for Laos).

### *Viral genome assembly and classification*

The output sequencing data (.fast5) from MinKNOW $_{\text{software}}$ was base-called with Guppy software using the High Accuracy (HAC) model to generate nucleotide sequence data for each fragment (reads) in the fastq format. These base-called data were then processed through the established workflow wf-artic on EPI2ME software to be assembled into consensus

sequences. Only reads with average Phred Quality (Q) score above 9 and minimum and maximum length of 250 and 1500 bps were used in the assembly process.

For viral classification - Consensus sequences from all sites were classified using the Pangolin tool (4.1.1) and Pangolin dataset (v1.14). Viral lineages were classified into eight categories corresponding to current and previous Variants of Concern (VOC): Delta, BA.1, BA.2, BA.2.75, BA.4, BA.5, XBB, and XBB.1.5-like. A lineage was classified as XBB.1.5-like based on the ECDC listing of Variants of Concern and includes lineages classified as XBB.1.5-like+F456L. All other XBB sublineages were classified as XBB.[3]

## S2: Bayesian hierarchical model

### ***Likelihood***

The general model likelihood takes the following form:

$$y_{i,j,t} \sim Student\left(\lambda,\ a_0 + a_i + a_{cov} + \gamma x_{i,j,t} + b_0 e^{b_i + b_{cov} + b_{T(i)}} t,\ \sigma^2\right)$$

where:

- $y_{i,j,t}$ is the log viral density (log10 genomes per mL) for the $j$-th swab of patient $i$ at time $t$.
- $T(i)$ is the randomized treatment allocation for individual $i$.
- $\sigma^2$ is the variance of the error in the fit and $\lambda$ is the degrees of freedom for the Student-t error model.
- $a_0$ and $b_0$ are the population mean intercept (baseline viral density) and slope (viral clearance rate), respectively.
- $a_i$ and $b_i$ are the individual random effects on the intercept and slope, respectively.
- $a_{cov}$ and $b_{cov}$ are the covariate effects on the intercept and slope.
- $x_{i,j,t}$ is human RNase P CT (scaled to have mean 0) for the $j$ swab from patient $i$ at time $t$, with a $\gamma$ parameter adjusting for the effect of human RNase P on the estimated viral density in the oropharyngeal eluates.

Covariate terms for the slope and intercept are: the reported days since symptom onset, study site, age, sex, and number of vaccine doses received. This model parameterised the treatment effect relative to a reference intervention (e.g. no study drug) as a proportional change ($e^{b_{T(i)}}$). As a sensitivity analysis, we parameterise it as an additive change as follows.

$$y_{i,j,t} \sim Student\left(\lambda,\ a_0 + a_i + a_{cov} + \gamma x_{i,j,t} + (b_0 e^{b_i + b_{cov}} + b_{T(i)})t,\ \sigma^2\right)$$

### ***Priors***

All models are fit using weakly informative priors on all parameters as follows.

- $a_0 \sim \text{Normal}(\text{mean} = 5, \text{sd} = 2)$
- $b_0 \sim \text{Normal}(\text{mean} = -0.5, \text{sd} = 1)$
- $\sigma \in [0, \infty) \sim \text{Normal}(\text{mean} = 1, \text{sd} = 1)$
- $\lambda \sim \text{Exponential}(1)$
- $a_{cov} \sim \text{Normal}(\text{mean} = 0, \text{sd} = 0.5)$
- $b_{cov} \sim \text{Normal}(\text{mean} = 0, \text{sd} = 0.5)$
- $b_{T(i)} \sim \text{Normal}(\text{mean} = 0, \text{sd} = 1)$
- $\gamma \sim \text{Normal}(\text{mean} = 0, \text{sd} = 1)$
- Individual random effects on the intercept $a_i$ and slope $b_i$ have a multivariate normal distribution with Cholesky parameterization as a prior, with location parameter $\mu = \begin{bmatrix} 0 \\ 0 \end{bmatrix}$ and standard deviation $\Sigma \sim \text{Exponential}(1)$ and correlation matrix for individual random effects $\Omega \sim \text{Cholesky}(2)$ as hyperparameters.

# S3: Supplementary figures

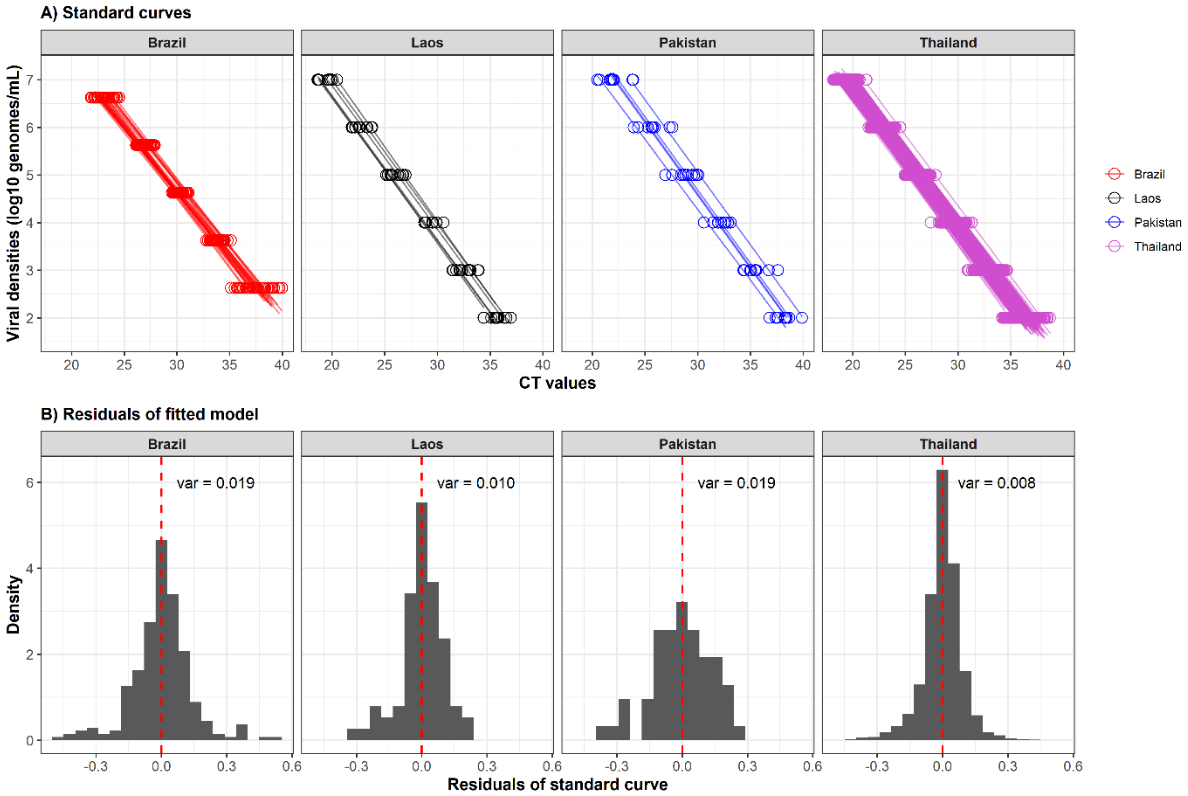


**Figure S1** Standard curves for the qPCR assay (Panel A) and the distributions of residuals of the mixed-effect linear regression model (Panel B) across all qPCR batches in 4 countries. Text annotations in Panel B represent variance of the residuals.

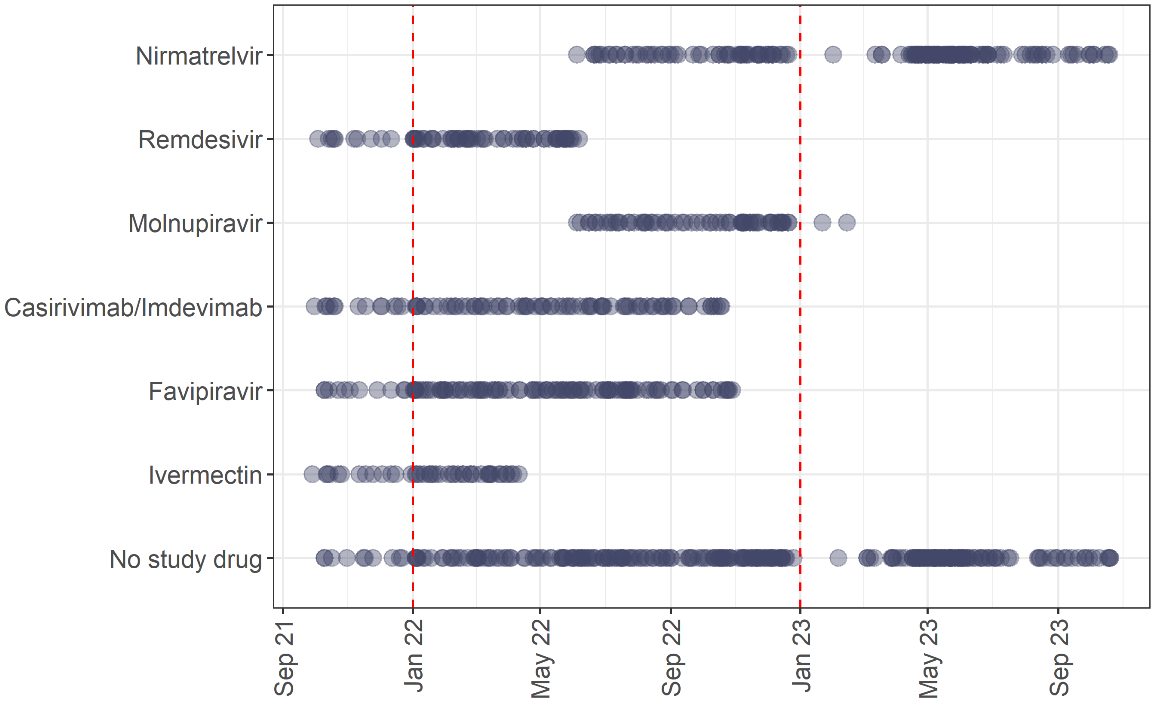


**Figure S2** Randomisation dates of the 800 patients included in the meta-analysis. Each semi-transparent circle represents one patient; therefore, the intensity of circles is proportional to the number of patients recruited on that date.

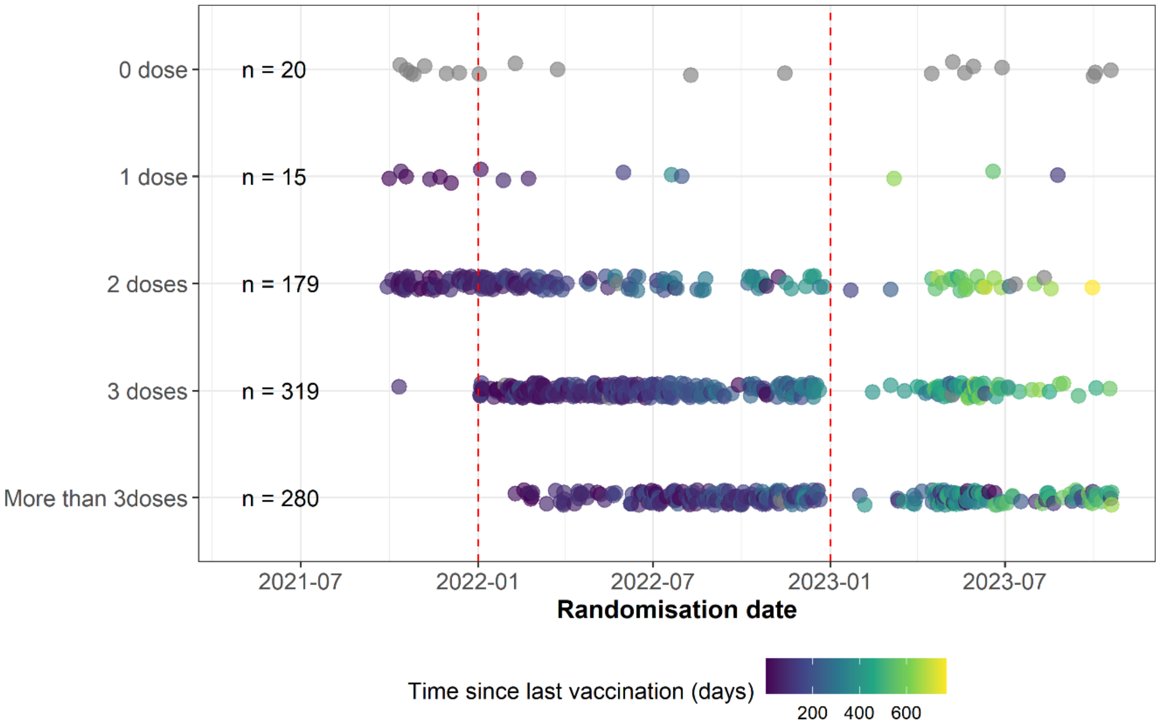


**Figure S3** Distribution of vaccination history of 800 included patients over time, classified by the number of vaccination doses. Points represent individual patients. Colors represent time since last vaccination (days), with grey indicating missing data or patients who had not received any vaccine.

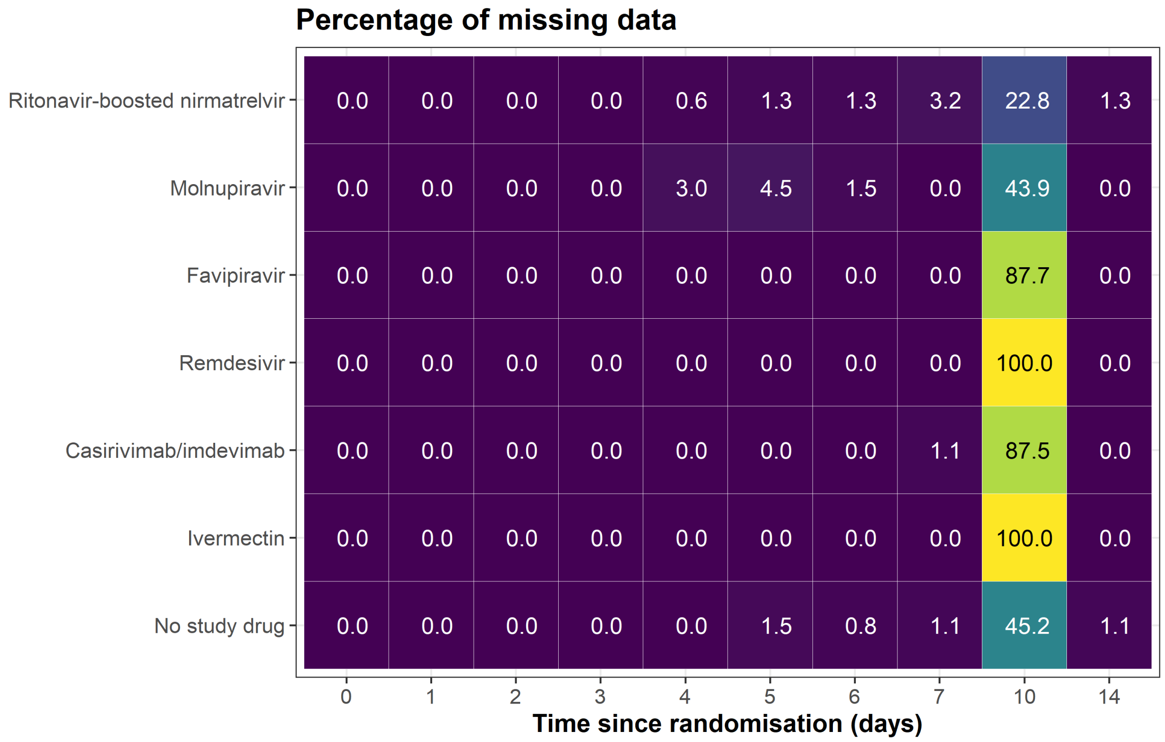


**Figure S4** Percentage of missing data by treatment arm and days since randomisation for 800 included patients. Day 10 swabs were introduced into the platform in August 2022; therefore, explaining the high proportion of missingness in early evaluated arms (100% in ivermectin and remdesivir).

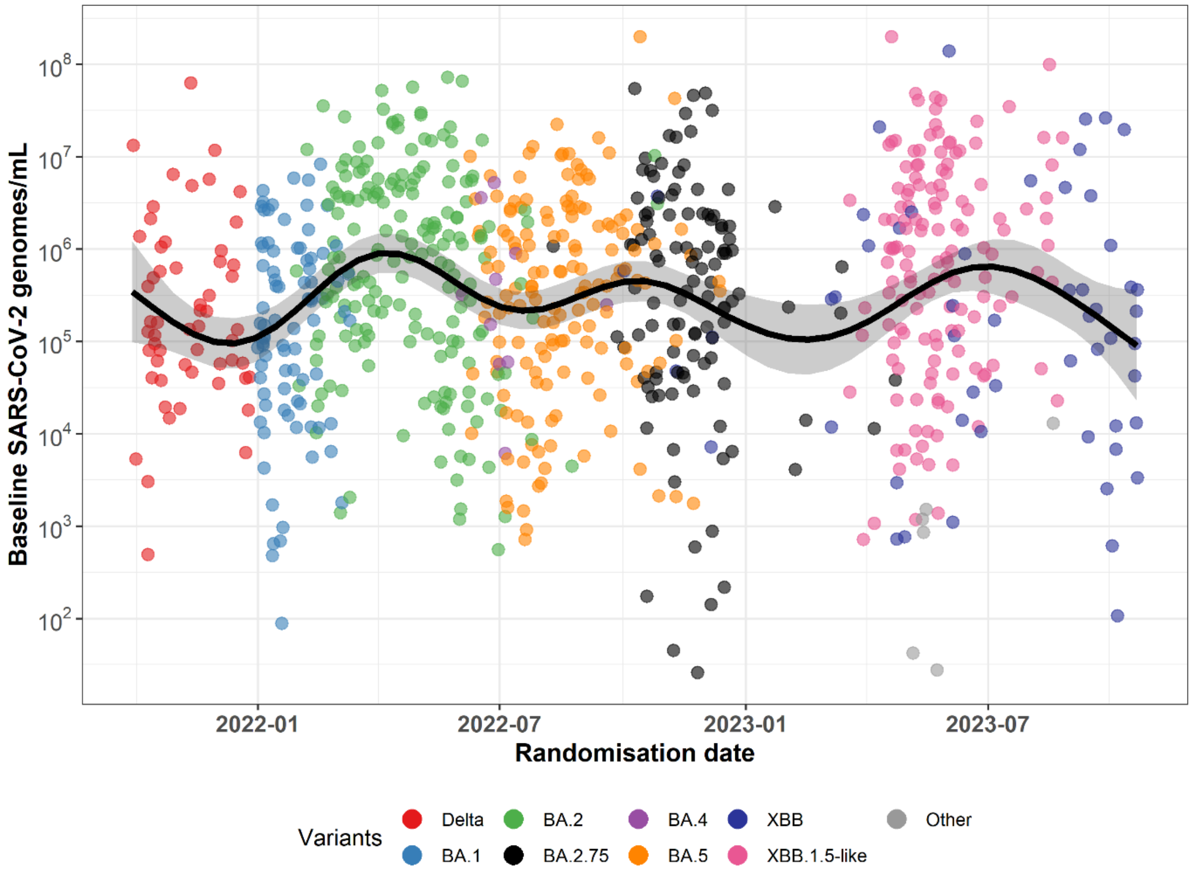


**Figure S5** Variation in the population baseline viral loads over time. The baseline viral load is taken at randomisation so it is not affected by the subsequent treatment. Colours show the main viral sub-lineages. The baseline viral load is defined as the geometric mean of the viral density (viral genomes) in eluates taken from four independent oropharyngeal swabs taken at randomisation.

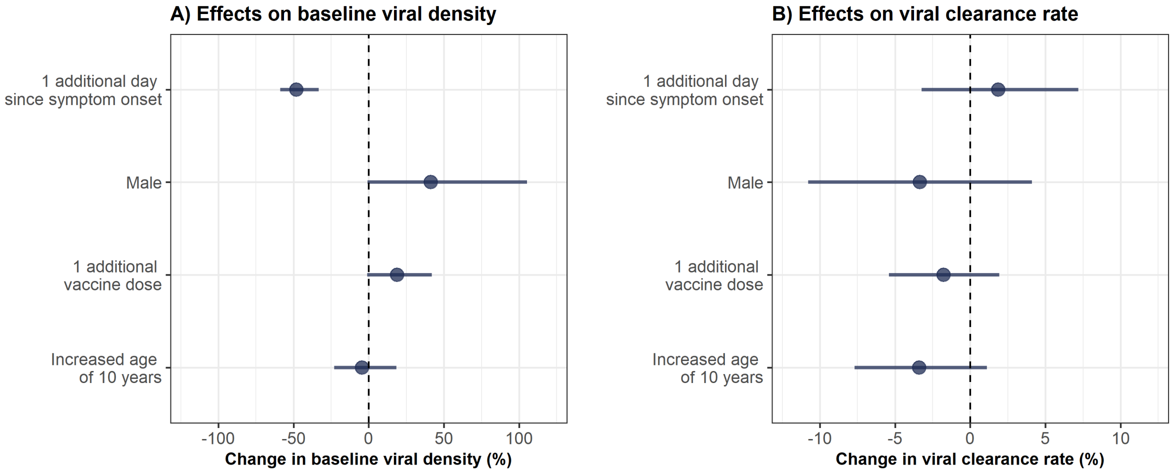


**Figure S6** Estimated effects of covariates (days since symptom onset, sex, number of vaccine doses, and age) on baseline viral densities and viral clearance rate between days 0 and 7. Points and error bars represent median and the 95% credible intervals, respectively.

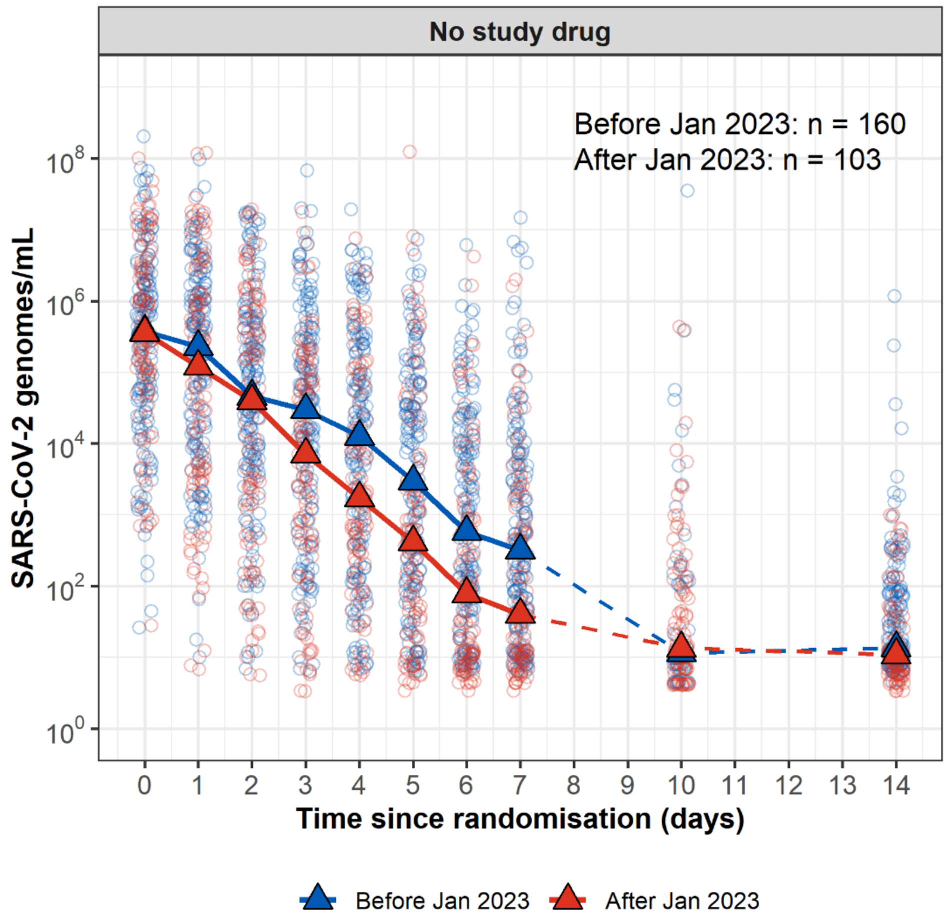


**Figure S7** Individual viral densities of 263 patients randomised to the no-study-drug arm before January 2023 (blue) and since January 2023 (red). Triangles show the daily median viral loads by arm.

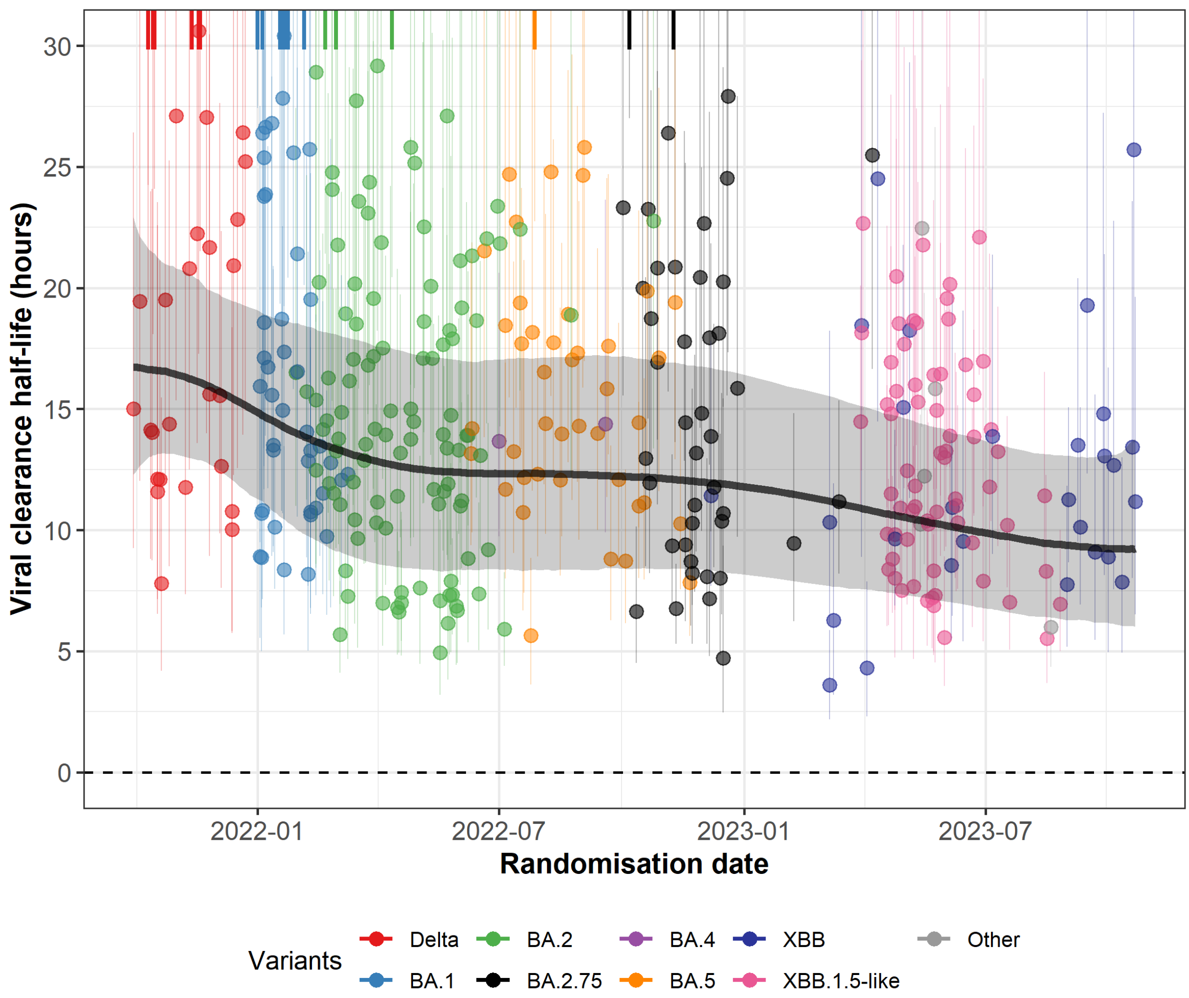


**Figure S8** Changes over time in the viral clearance half-lives estimated between days 0 and 7 ($\alpha_{0\text{-}7}$). The median (95% credible interval) population viral clearance half-life is shown by the black line (grey area). Individual median clearance half-life estimates (95% CrI) are shown for patients in the no study drug, ivermectin or favipiravir arms (ineffective interventions). Colours show the main viral sub-lineages. The Y-axis was truncated at 30 hours, with ticks on the top axis indicating the timing and viral variant of patients with clearance half-lives greater than 30 hours.

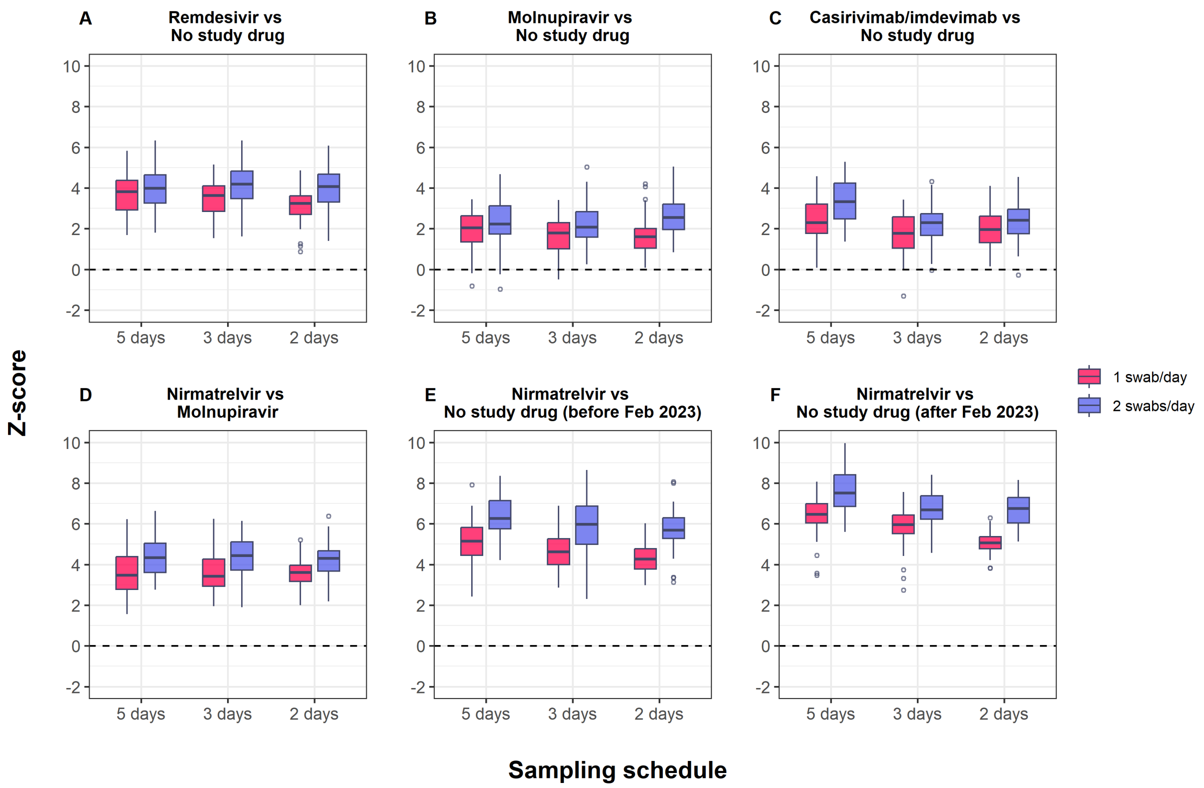


**Figure S9** Bootstrap z-scores for six treatment effects (each with a sample size of n=50 per arm), broken down by sampling schedule and the number of oropharyngeal swab samples taken per day. 5 days: samples taken on days 0 to 4; 3 days: samples taken on days 0, 2, and 4; 2 days: samples taken on days 0 and 4. Boxplots show the median and interquartile range for the bootstrapped datasets. The comparisons only use concurrently randomised controls.

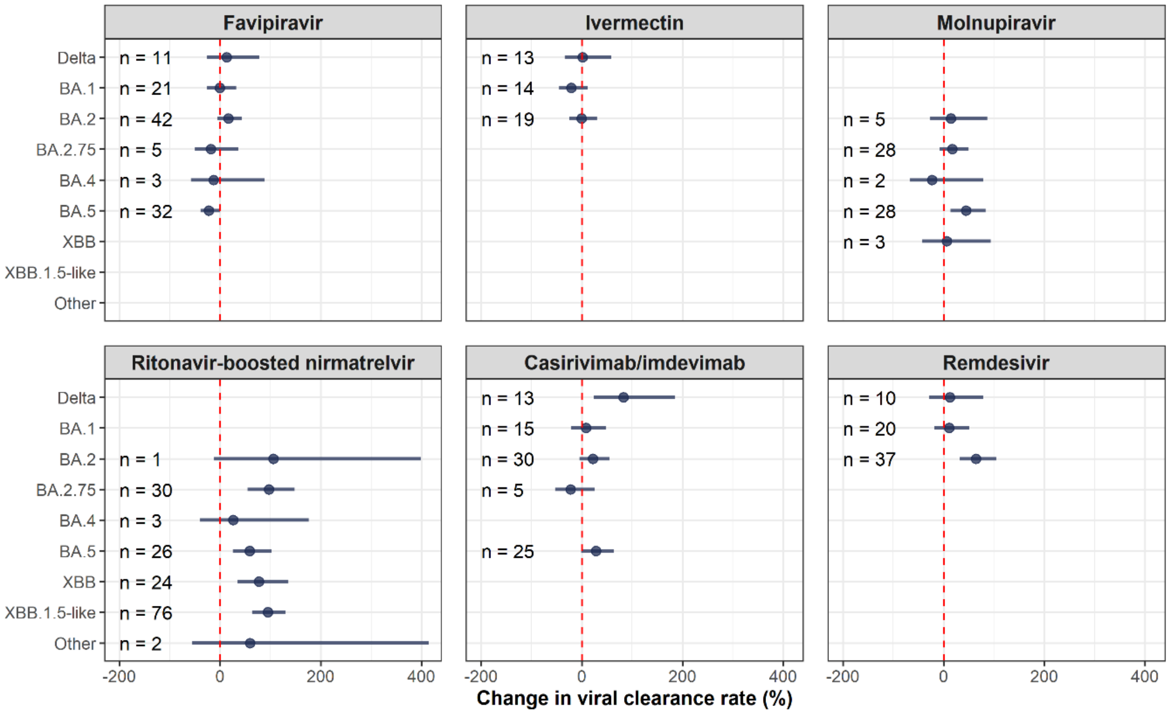


**Figure S10** Estimated treatment effects as percentage changes in viral clearance rate of intervention drugs by viral variants. Points and error bars represent median treatment effects and 95% credible intervals, respectively. Text annotations represent the number of patients in each subgroup. The effects of covariates were estimated using the values over Day 0 to Day 7.

## S4: Supplementary tables

**Table S1** The estimated viral clearance rate and viral clearance half-life at the beginning and end of the randomisation of each treatment arm, along with their relative reduction in viral clearance half-life.

| Treatment arm | Start | | | End | | | Relative reduction in half-life[c] |
|---|---|---|---|---|---|---|---|
| | Date | Rate[a] | Half-life[b] | Date | Rate[a] | Half-life[b] | |
| No study drug | Sep 2021 | -0.43 (-0.47; -0.40) | 16.6 (15.3; 18.2) | Oct 2023 | -0.78 (-0.90; -0.68) | 9.2 (8.0; 10.6) | 44% (19; 64%) |
| Ritonavir-boosted nirmatrelvir | Jun 2022 | -1.12 (-1.27; -0.99) | 6.4 (5.7; 7.3) | Oct 2023 | -1.5 (-1.74; -1.30) | 4.8 (4.2; 5.5) | 26% (-4; 42%) |
| Ivermectin | Sep 2021 | -0.35 (-0.40; -0.32) | 20.4 (18.2; 22.8) | Apr 2022 | -0.47 (-0.54; -0.41) | 15.5 (13.5; 17.6) | 23% (3; 51%) |
| Casirivimab/ imdevimab | Oct 2021 | -0.50 (-0.55; -0.45) | 14.4 (13.1; 15.9) | Oct 2022 | -0.69 (-0.78; -0.61) | 10.5 (9.3; 11.9) | 26% (1; 51%) |
| Remdesivir | Oct 2021 | -0.54 ( -0.59; -0.49) | 13.5 (12.2; 14.9) | Jun 2022 | -0.72 (-0.83; -0.63) | 10.0 (8.7; 11.4) | 25% (1; 51%) |
| Favipiravir | Oct 2021 | -0.40 (-0.44; -0.37) | 17.9 (16.5; 19.5) | Oct 2022 | -0.55 (-0.63; -0.49) | 13.1 (11.5; 14.8) | 27% (2; 49%) |
| Molnupiravir | Jun 2022 | -0.75 (-0.86; -0.65) | 9.7 (8.4; 11.1) | Jan 2023 | -0.79 (-0.91; -0.70) | 9.1 (8.0; 10.3) | 6% (-11; 17%) |

[a] The values are represented as median viral clearance rate in $log_{10}$ genomes per mL per day (interquartile range); [b] The values are represented as median viral clearance half-life in hours (interquartile range); [c] The values are represented as median relative reduction in viral clearance half-life (95% credible interval).

**Table S2** The estimated treatment effect of intervention drugs as changes in viral clearance rate relative to no study drug arm.

| Treatment drug | Follow-up duration | Changes in viral clearance rate relative to no study drug arm (%) | | |
|---|---|---|---|---|
| | | Median estimate | 80% credible inverval | 95% credible interval |
| Ivermectin | 5 days | -21% | -31 to -9% | -36 to -2% |
| Ivermectin | 7 days | -18% | -27 to -7% | -32 to -1% |
| Casirivimab/Imdevimab | 5 days | 36% | 23 to 49% | 16 to 58% |
| Casirivimab/Imdevimab | 7 days | 16% | 7 to 27% | 2 to 33% |
| Remdesivir | 5 days | 48% | 32 to 64% | 25 to 75% |
| Remdesivir | 7 days | 24% | 12 to 37% | 7 to 44% |
| Favipiravir | 5 days | -5%% | -13 to 4% | -18 to 9% |
| Favipiravir | 7 days | -7% | -14 to 1% | -17 to 5% |
| Molnupiravir | 5 days | 47% | 32 to 62% | 25 to 71% |
| Molnupiravir | 7 days | 28% | 16 to 41% | 11 to 48% |
| Ritonavir-boosted nirmatrelvir | 5 days | 127% | 110 to 144% | 102 to 155% |
| Ritonavir-boosted nirmatrelvir | 7 days | 92% | 80 to 106% | 73 to 113% |

## S5: Inclusion-exclusion criteria

The entry criteria were: (i) SARS-CoV-2 positive as defined either as a nasal lateral flow antigen test that became positive within 2 minutes (STANDARD Q COVID-19 Ag Test, SD Biosensor, Suwon-si, South Korea) or a positive PCR test with a cycle threshold value less than 25 (all viral gene targets) within the previous 24 h (both these tests ensure the majority of recruited patients have high viral densities); (ii) reported symptoms of COVID-19 for less than 4 days (<96 h); (iii) oxygen saturation on room air ≥96% measured by pulse oximetry at the time of screening; (iv) unimpeded in activities of daily living; (v) agreed to adhere to all procedures, including availability and contact information for follow-up visits.

Exclusion criteria included taking any concomitant medications or drugs, chronic illness or condition requiring long-term treatment or other clinically significant comorbidity, laboratory abnormalities at screening (haemoglobin <8 g/dL, platelet count <50 000/µL, abnormal liver function tests, and estimated glomerular filtration rate <70 mL/min per 1·73 $m^2$), pregnancy (a urinary pregnancy test was performed in females), actively trying to become pregnant, lactation, contraindication or known hypersensitivity to any of the proposed therapeutics, currently participating in a COVID-19 therapeutic or vaccine trial, or evidence of pneumonia (although imaging was not required). After a detailed explanation of study procedures and requirements all patients provided fully informed written consent.

## S6: Ethical approvals

In Thailand the trial was approved by the Faculty of Tropical Medicine Ethics Committee, Mahidol University, (reference Certificate of Approval number MUTM 2021-057-03); in Brazil by the Research Ethics Committee of the Universidade Federal de Minas Gerais (COEP-UFMG, Minas Gerais, Brazil, COEP-UFMG) and National Research Ethics Commission- (CONEP, Brazil, COEP-UFMG and CONEP Ref: CAAE:51593421.1.0000.5149); in Laos by the National Ethics Committee for Health Research (NECHR, Lao People's Democratic Republic, Submission ID 2022.48) and the Food & Drugs Department (FDD, Lao People's Democratic Republic, 13066/FDD_12Dec2022); in Pakistan by the National Bioethics Committee (NBC No.4-87/COVID-111/22/842) the Ethics Review Committee (ERC 2022-7496-21924) and the Drug Regulatory Authority (DRAP Ref: No.0318/2022-CT (PS)).

# S7: List of Sites and Investigators (PLATCOV Collaborative Group)

## Sites

1. Hospital for Tropical Diseases (HTD), Faculty of Tropical Medicine, Mahidol University, 420/6 Rajvithi Road, Bangkok, 10400, Thailand
2. Universidade Federal de Minas Gerais, Av. Antônio Carlos, 6627 Belo Horizonte, Minas Gerais 31270 – 901, Brazil
3. Mahosot Hospital, Quai Fa Ngum, Vientiane, Laos
4. Aga Khan University, National Stadium Rd, Karachi, Pakistan

**Co-principal investigators:**

Nicholas J White (nickw@tropmedres.ac)[1,2]
William HK Schilling (william@tropmedres.ac)[1,2]

**Thailand: Faculty of Tropical Medicine, Mahidol University**

**Site and Country Principal investigator:**

Weerapong Phumratanaprapin[3]

**Accountable Investigator:**

Viravarn Luvira[3]

**Co-Investigators/team members:**

James J Callery[1,2]
Nicholas PJ Day[1,2]
Sasithon Pukrittayakamee[1,3]
Simon Boyd[1,2]
Cintia Cruz[1,2]
Arjen M Dondorp[1,2]
Walter RJ Taylor[1,2]
James A Watson[1,4]
Phrutsamon Wongnak [1,2]
Watcharapong Piyaphanee[3]
Kittiyod Poovorawan[1,3]
Thundon Ngamprasertchai[5]
Tanaya Siripoon[3]
Borimas Hanboonkunupakarn[1,3]
Kesinee Chotivanich[1,3]
Podjanee Jittamala[1,5]
Mallika Imwong[1,6]
Maneerat Ekkapongpisit[1]
Varaporn Kruabkontho[1]

Thatsanun Ngernseng[1]
Jaruwan Tubprasert[1]
Mohammad Yazid Abdad[1,2]
Srisuda Keayarsa[3]
Orawan Anunsittichai[1]
Maliwan Hongsuwan[1]
Yutatirat Singhaboot[3]
Wanassanan Madmanee[1]
Elizabeth M Batty[1,2]
Runch Tuntipaiboontana[1]
Watcharee Pagornrat[1]
Amornrat Promsongsil[1]
Shivani Singh[1,2]
Manisaree Saroj[1]
Jindarat Kouhathong [1]
Kanokon Suwannasin[1]
Ellen Beer[1]
Tanatchakorn Asawasriworanan[1]
Stuart Blacksell[1,2]
Salwaluk Panapipat[1]
Naomi Waithira[1,2]
Joel Tarning[1,2]
Nuttakan Tanglakmankhong[1]

**Thailand: Bangplee Hospital (**(discontinued)
**Site Principal investigator:**
Pongtorn Hanboonkunupakarn [6]
**Co-investigator:**
Sakol Sookprome[6]

**Thailand: Vajira Hospital** (discontinued)
**Site Principal investigator:**
Vasin Chotivanich[8]
**Co-investigators:**
Wiroj Ruksakul[8]
Chunlanee Sangketchon[9]

**Brazil: Universidade Federal de Minas Gerais**
**Site and Country Principal investigator:**
Mauro M Teixeira[10]

**Co-Investigators:**
Lisia M Esper[10]
Fernando R Ascencao[11]
Renato S Aguiar[12]
Pedro J Almeida[10]

**Laos: Mahosot Hospital**
**Site Principal investigator:**
Elizabeth Ashley [2,13]
**Co-Investigators:**
Audrey Dubot-Pérès[2,13, 14]
Mayfong Mayxay [2,13,15]
Manivanh Vongsouvath[16]
Danoy Chommanam[13]
Latsaniphone Boutthasavong [13]
Vayouly Vidhamaly[13]
Koukeo Phommasone[13]
Terry John Evans[2,13]
Susath Vongphachanh [17]
Sisouphanh Vidhamaly[18]
Ammala Chingsanoon [18]
Sixiong Bisayher [18]

**Pakistan: Aga Khan Hospital**
**Site and Country Principal investigator:**
M Asim Beg [19]
**Co-Investigators:**
Abdul Momin Kazi[19]
Farah Qamar[19]
Najia K Ghanchi[19]
Syed Faisal Mahmood [19]

**Thailand: Ministry of Public Health**
Manus Potaporn[20]
Attasit Srisubat[20]
Bootsakorn Loharjun [20]

**Affiliations:**
1. Mahidol Oxford Tropical Medicine Research Unit, Faculty of Tropical Medicine, Mahidol University, Bangkok, Thailand

2. Centre for Tropical Medicine and Global Health, Nuffield Department of Medicine, Oxford University, Oxford, UK
3. Department of Clinical Tropical Medicine, Faculty of Tropical Medicine, Mahidol University, Bangkok, Thailand
4. Oxford University Clinical Research Unit, Vietnam
5. Department of Clinical Tropical Hygiene, Faculty of Tropical Medicine, Mahidol University, Bangkok, Thailand
6. Department of Molecular Tropical Medicine and Genetics, Faculty of Tropical Medicine, Mahidol University, Bangkok, Thailand
7. Bangplee Hospital, Ministry of Public Health, Samut Prakarn province, Thailand
8. Faculty of Medicine, Navamindradhiraj University, Bangkok, Thailand
9. Faculty of Science and Health Technology, Navamindradhiraj University, Bangkok, Thailand
10. Clinical Research Unit, Center for Advanced and Innovative Therapies, Universidade Federal de Minas Gerais, Brazil
11. Department of Biochemistry and Immunology, Universidade Federal de Minas Gerais, Brazil
12. Department of Genetics, Ecology and Evolution, Institute of Biological Sciences, Universidade Federal de Minas Gerais, Brazil
13. Lao-Oxford-Mahosot Hospital-Wellcome Trust Research Unit, Microbiology Laboratory, Mahosot Hospital, Vientiane, Lao P.D.R.
14. Unité des Virus Émergents, Marseille, France
15. Institute for Research and Education Development, University of Health Sciences, Vientiane, Lao P.D.R.
16. Microbiology Laboratory, Mahosot Hospital, Vientiane, Lao P.D.R.
17. Mahosot Hospital, Vientiane, Lao P.D.R.
18. Pulmonology Department, Mahosot Hospital, Vientiane, Lao P.D.R.
19. Aga Khan University, Karachi, Pakistan
20. Department of Medical Services, Ministry of Public Health, Nonthaburi, Thailand

# S8: Master protocol of the PLATCOV trial
